# New mud dragons from Svalbard: three new species of *Cristaphyes* and the first Arctic species of *Pycnophyes* (Kinorhyncha: Allomalorhagida: Pycnophyidae)

**DOI:** 10.7717/peerj.5653

**Published:** 2018-09-28

**Authors:** Martin Vinther Sørensen, Katarzyna Grzelak

**Affiliations:** 1 Natural History Museum of Denmark, University of Copenhagen, Copenhagen, Denmark; 2 Faculty of Biology and Environmental Protection, Laboratory of Polar Biology and Oceanobiology, University of Łódź, Łódź, Poland; 3 Department of Marine Ecology, Institute of Oceanology PAN, Sopot, Poland

**Keywords:** Kinorhynchs, *Cristaphyes*, Pycnophyes, Meiofauna, Arctic

## Abstract

**Background:**

Kinorhynchs are marine, microscopic invertebrates inhabiting the seafloors. Their segmented trunk equipped with spines and processes has inspired scientists to give them the common name “mud dragons.” Even though kinorhynchs have been known since the 19th century, less than 300 species are known to science, and it is still considered a largely understudied animal group—in particular in the Arctic, from which only 23 species are known so far.

**Methods:**

Samples were collected at eight stations around Svalbard and in the fjords of Spitsbergen. Meiofauna was extracted from the sediment cores with LUDOX centrifugation method, and kinorhynchs were picked up and mounted for light- and scanning electron microscopy.

**Results:**

Four new species of the kinorhynch family Pycnophyidae are described from Svalbard: *Cristaphyes dordaidelosensis* sp. nov., *C. glaurung* sp. nov., *C. scatha* sp. nov., and *Pycnophyes ancalagon* sp. nov. The new species are generally recognized by their distribution of setae along the trunk segments.

**Discussion:**

After the discovery of the new species, Pycnophyidae becomes with 14 species the most diverse kinorhynch genus in the Arctic, closely followed by Echinoderidae with 13 species. So far, these are the only kinorhynch families with an Arctic distribution.

## Introduction

The kinorhynch fauna of Svalbard has recently been explored during annual meiofauna samplings in the fjords and surrounding waters, through a period from 2013 to 2017. The studies have so far resulted in the description of four new species of *Echinoderes* (Grzelak & Sørensen, 2018, in press), a redescription of *Echinoderes arlis* Higgins, 1966 (Grzelak & Sørensen, in press), and the finding of four species of *Echinoderes* that otherwise are only known from West Greenland and Eastern Canada (Grzelak & Sørensen, 2018, in press). The latter finding, together with the record of the Alaskan species *E. arlis*, prompted (Grzelak & Sørensen, in press) to suggest that Arctic *Echinoderes* species may show a circumpolar distribution.

So far, the results from the Svalbard surveys have focused on species of Echinoderidae only. The present contribution, which is also expected to be the last taxonomic contribution in the series, focuses on species of Pycnophyidae. So far, only two pycnophyid species are known from Svalbard: *Krakenella mokievskii* (Adrianov, 1995) and *K. spitsbergensis* (Adrianov, 1995). Both species were described from Isfjorden on the east coast of Spitsbergen (Adrianov, 1995). Additional Arctic species of *Krakenella* includes *K. barentsi* (Adrianov, 1999 in Adrianov & Malakhov, 1999) and *K. galtsovae* (Adrianov, 1999 in Adrianov & Malakhov, 1999) from the nearby Barents Sea, *K. borealis* (Higgins & Korczynski, 1989) and *K. canadensis* (Higgins & Korczynski, 1989) from NW Territory in Canada, and *K. greenlandica* (Higgins & Kristensen, 1988) from Disko Island, West Greenland (Higgins & Kristensen, 1988; Higgins & Korczynski, 1989; Adrianov & Malakhov, 1999). Otherwise, *Cristaphyes* is the only other pycnophyid genus known from the Arctic. Records of *Cristaphyes* include: *Cristaphyes arctous* (Adrianov, 1999 in Adrianov & Malakhov, 1999) from the Fram Strait and a locality NE of Svalbard, *C. chukchiensis* (Higgins, 1991) from the Chukchi Sea off Alaska, and *C. cryopygus* (Higgins & Kristensen, 1988) from Disko Island, West Greenland (Higgins & Kristensen, 1988; Higgins, 1991; Adrianov & Malakhov, 1999).

The present contribution describes three additional new Arctic species of *Cristaphyes*, and the first Arctic species of *Pycnophyes*. We also summarize previous works reporting Pycnophyidae family in the area and provide distribution ranges of all Pycnophyidae species recorded in the Arctic region. Our study represents an additional step to unveil the diversity of mud dragons in the Arctic and provide evidence that the diversity of kinorhynch fauna is still far from being known.

## Materials and Methods

**Figure 1:**
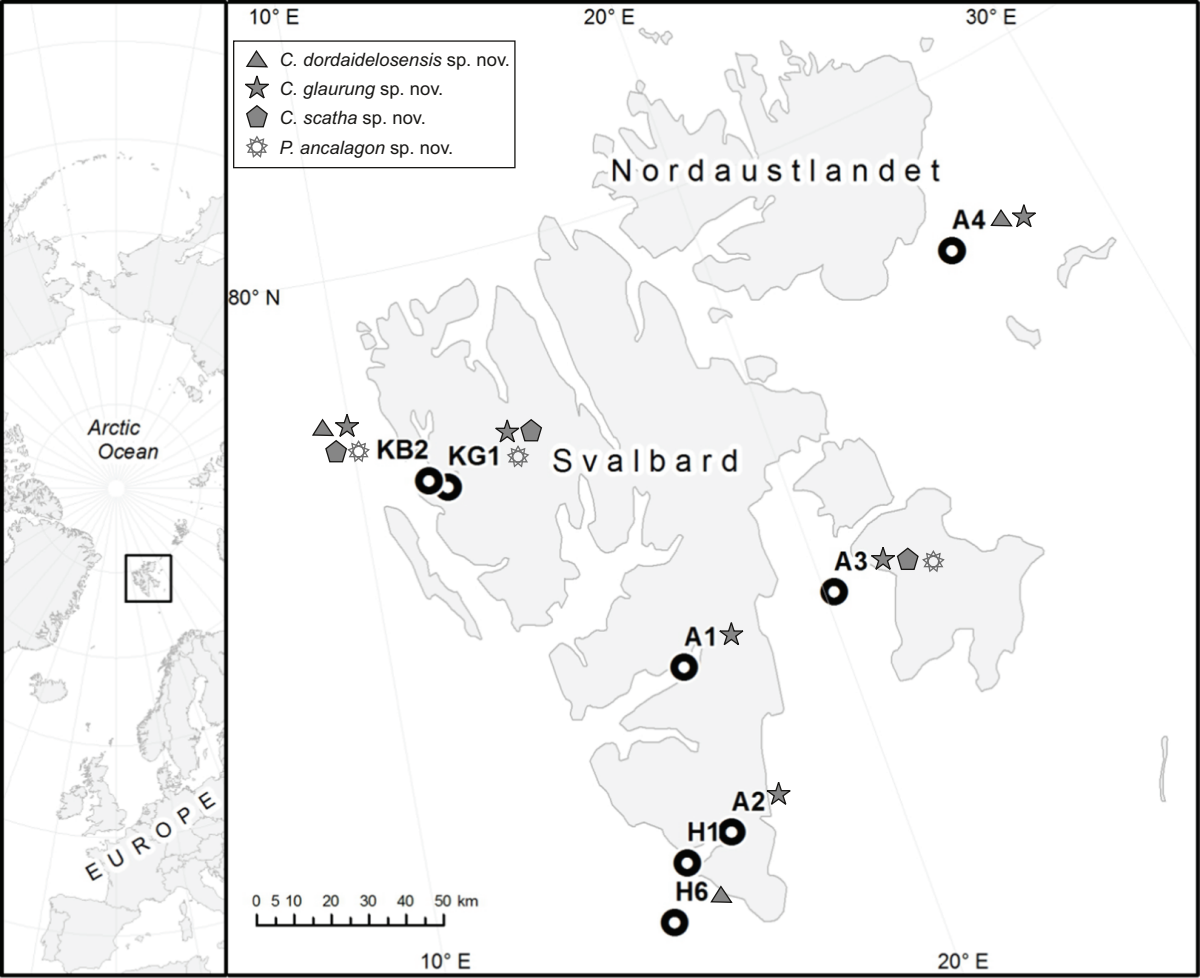
Map showing the sampling stations around Svalbard. See Table 1 for detailed data on stations.

Samples were collected in the European sector of the Arctic Ocean during two cruises: (1) in July–August 2013 on board of the *R/V Oceania* in Hornsund (SW Spitsbergen) and (2) in May 2016 on board of the R/V *Helmer Hanssen* in Hornsund, Van Mijenfjorden (SW Spitsbergen), Kongsfjorden (NW Spitsbergen), and east of Spitsbergen (Table 1; Fig. 1). At stations H1 and H6 in Hornsund and KG1, KB2 in Kongsfjorden, samples were taken with a Niemistö gravity corer (nine cm inner diameter). Three cores obtained from separate deployments were sampled for meiofaunal analyses using a Plexiglas tube with an inner diameter of 3.6 cm. At the remaining stations, samples were taken with a giant box corer, and three subsamples were collected from each deployment using the same Plexiglas tube. The upper five cm of sediment from each subsample were taken and fixed in a 4% formaldehyde solution in seawater buffered with borax. The fixed samples were subsequently washed with freshwater in a 32 μm sieve, and meiofauna organisms were extracted using centrifugation method, with a solution of colloidal silica LUDOX TS50 (Vincx, 1996). All meiofaunal organisms were counted and classified at higher taxonomic levels under a Nikon SMZ1500 stereomicroscope after staining with Bengal Rose to facilitate sorting process. After sorting, kinorhynchs were picked out and stored in a 4% formaldehyde solution.

**Table 1:** Summary of data on stations, species identities, and catalogue numbers.

Station	Location	Date	Position	Depth	Species	Mounting	Type status and catalogue numbers
A1	Van Mijenfjorden	May 19, 2016	77^°^49.74′N 016^°^28.38′E	59 m	*C. glaurung* sp. nov.	LM	1♂ paratype, NHMD-233057
A2	Hornsund	May 20, 2016	77^°^01.21′N 016^°^27.29′E	120 m	*C. glaurung* sp. nov.	LM	1♀ paratype, NHMD-233056
A3	Storfjorden	May 21, 2016	77^°^56.61′N 020^°^13.10′E	96 m	*C. glaurung* sp. nov.	LM	1♀ paratype, NHMD-233055
						SEM	3♀, 1♂ non-types
					*C. scatha* sp. nov.	SEM	1♀ non-type
					*P. ancalagon* sp. nov.	SEM	2♀, 1♂ non-types
A4	S. of Nordaustlandet	May 24, 2016	79^°^12.53′N 025^°^59.74′E	217 m	*C. dordaidelosensis* sp. nov.	SEM	1♂ non-type
					*C. glaurung* sp. nov.	LM	♀ holotype, NHMD-233053, 1♀ paratype, NHMD-233054
						SEM	1♀ non-type
H1	Hornsund	July 27, 2013	76^°^56.31′N 015^°^22.56′E	155 m	*Pycnophyes* sp. 1	SEM	1♀ non-type
H6	Hornsund	Aug. 2, 2013	76^°^40.98′N 014^°^48.73′E	236 m	*C. dordaidelosensis* sp. nov.	LM	♂ holotype, NHMD-233049
KG1	Kongsfjorden	Aug. 7, 2013	78^°^55.85′N 012^°^08.37′E	105 m	*C. glaurung* sp. nov.	SEM	2♀, 1♂ non-types
					*C. scatha* sp. nov.	LM	1♂ holotype, NHMD-233061, 1♀ paratype NHMD-233062
					*P. ancalagon* sp. nov.	LM	♀ holotype, NHMD-233064, 4♀, 2♂ paratypes, NHMD-233065-233070
						SEM	1♀, 1♂ non-types
KB2	Kongsfjorden	Aug. 5, 2013	78^°^58.69′N 011^°^42.79′E	310 m	*C. dordaidelosensis* sp. nov.	LM	♂ Paratype, NHMD-233050
					*C. glaurung* sp. nov.	LM	1♀, 1♂ paratypes, NHMD-233058-233059
					*C. scatha* sp. nov.	LM	1♀ paratype, NHMD-233063
					*P. ancalagon* sp. nov.	LM	1♀, 1♂ paratypes, NHMD-233071-233072

Specimens for light microscopy (LM), were dehydrated through a graded series of water and glycerin and mounted in Fluoromount-G. The specimens were examined using an Olympus BX51 microscope (University of Copenhagen) and a Nikon E600 (Institute of Oceanology, Sopot) microscope, both equipped with differential interference contrast optics. The microphotographic documentation was done using an Olympus DP27 camera, and measurements were made with Cell^D software. All obtained dimensions reported in the tables are based on mounted LM specimens. Specimens for scanning electron microscopy (SEM) were dehydrated through a graded alcohol–acetone series and critical point dried. Dried specimens were mounted on aluminum stubs, sputter coated with platinum–palladium mix and examined with a JEOL JSM-6335F Field Emission scanning electron microscope. Line art figures were made with Adobe Illustrator CS6, based on imported LM micrographs, and supplemented with information obtained with SEM.

The electronic version of this article in portable document format will represent a published work according to the International Commission on Zoological Nomenclature (ICZN), and hence the new names contained in the electronic version are effectively published under that Code from the electronic edition alone. This published work and the nomenclatural acts it contains have been registered in ZooBank, the online registration system for the ICZN. The ZooBank LSIDs (Life Science Identifiers) can be resolved and the associated information viewed through any standard web browser by appending the LSID to the prefix http://zoobank.org/. The LSID for this publication is: urn:lsid:zoobank.org:pub:72D489B2-E8B6-499B-A0C1-10BCCF5E8A29. The online version of this work is archived and available from the following digital repositories: PeerJ, PubMed Central, and CLOCKSS.

## Systematic Account

**Figure 2:**
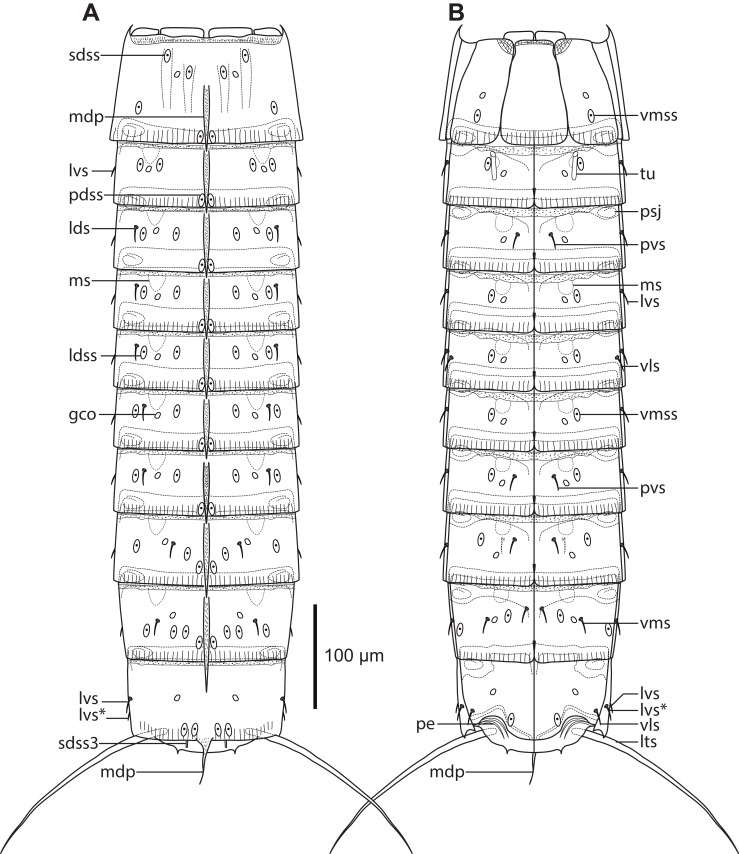
Line art illustrations of *Cristaphyes dordaidelosensis* sp. nov. (A) Male, dorsal view. (B) Male, ventral view. Abbreviations: gco, glandular cell outlet; lds, laterodorsal seta; ldss, laterodorsal sensory spot; lts, lateral terminal spine; lvs, lateroventral seta, * marks the additional lateroventral seta on segment 10; mdp, middorsal process; ms, muscular scar; pdss, paradorsal sensory spot; pe, penile spines; psj, peg-and-socket joint; pvs, paraventral seta; sdss, subdorsal sensory spot; sdss3, subdorsal sensory spot type 3; tu, tube; vls, ventrolateral seta; vms, ventromedial seta; vmss, ventromedial sensory spot. Setae drawn with dashed lines indicate alternative position of setae showing intraspecific variation.

**Figure 3:**
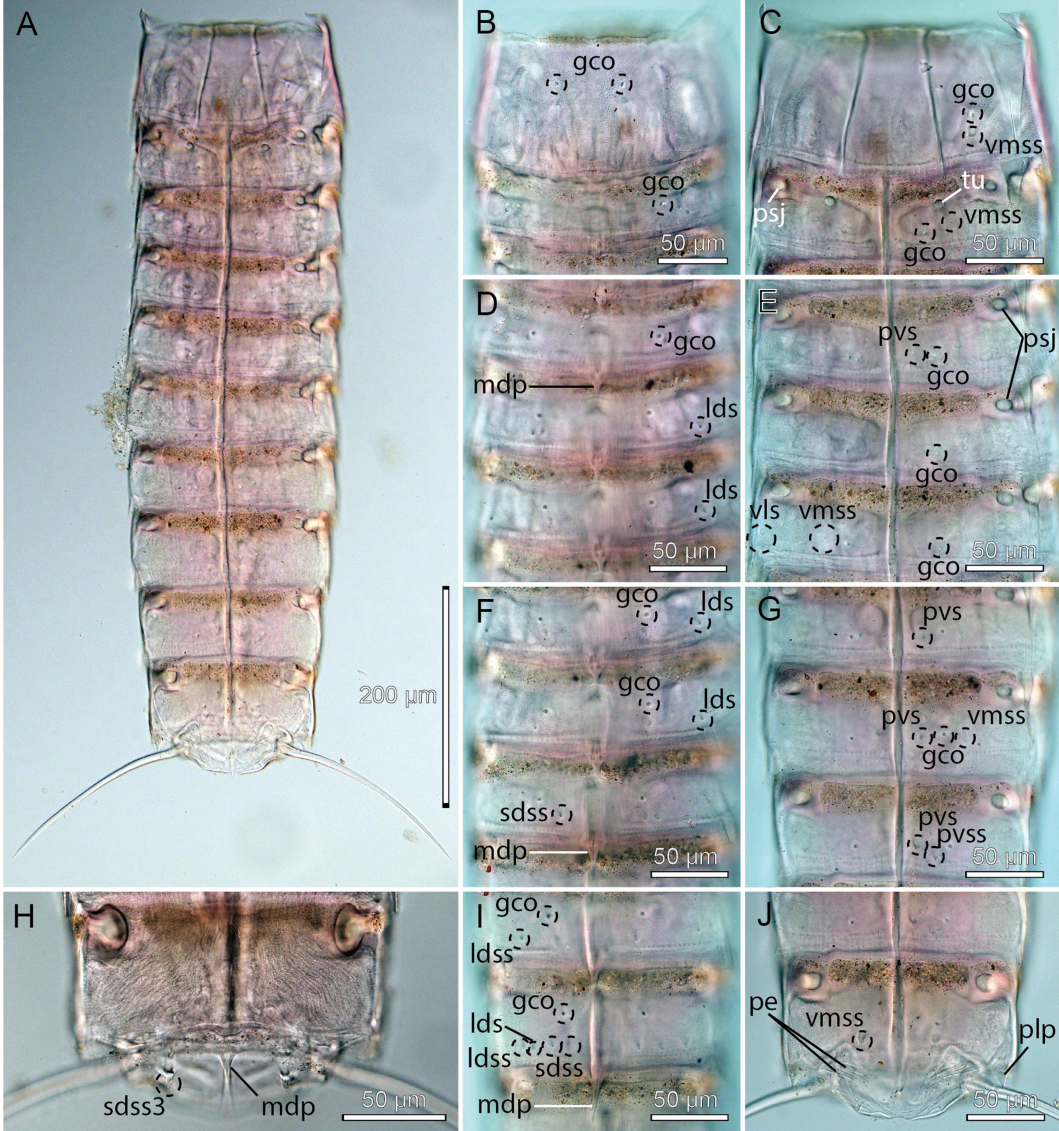
Light micrographs showing overviews and details of male holotype, NHMD-233049, of *Cristaphyes dordaidelosensis* sp. nov. (A) Ventral overview. (B) Segments 1–2, dorsal view. (C) Segments 1–2, ventral view. (D) Segments 3–5, dorsal view. (E) Segments 3–5, ventral view. (F) Segments 5–7, dorsal view. (G) Segments 7–9, ventral view. (H) Segments 10–11, focused at middorsal process of segment 10; note the spermatozoa that completely fills segment 10. (I) Segments 8–9, dorsal view. (J) Segments 9–11, ventral view. Abbreviations: gco, glandular cell outlet; lds, laterodorsal seta; ldss, laterodorsal sensory spot; mdp, middorsal process; pe, penile spines; plp, posterolateral process; psj, peg-and-socket joint; pvs, paraventral seta; pvss, paraventral sensory spot; sdss, subdorsal sensory spot; sdss3, subdorsal sensory spot type 3; tu, tube; vls, ventrolateral seta; vmss, ventromedial sensory spot.

**Figure 4:**
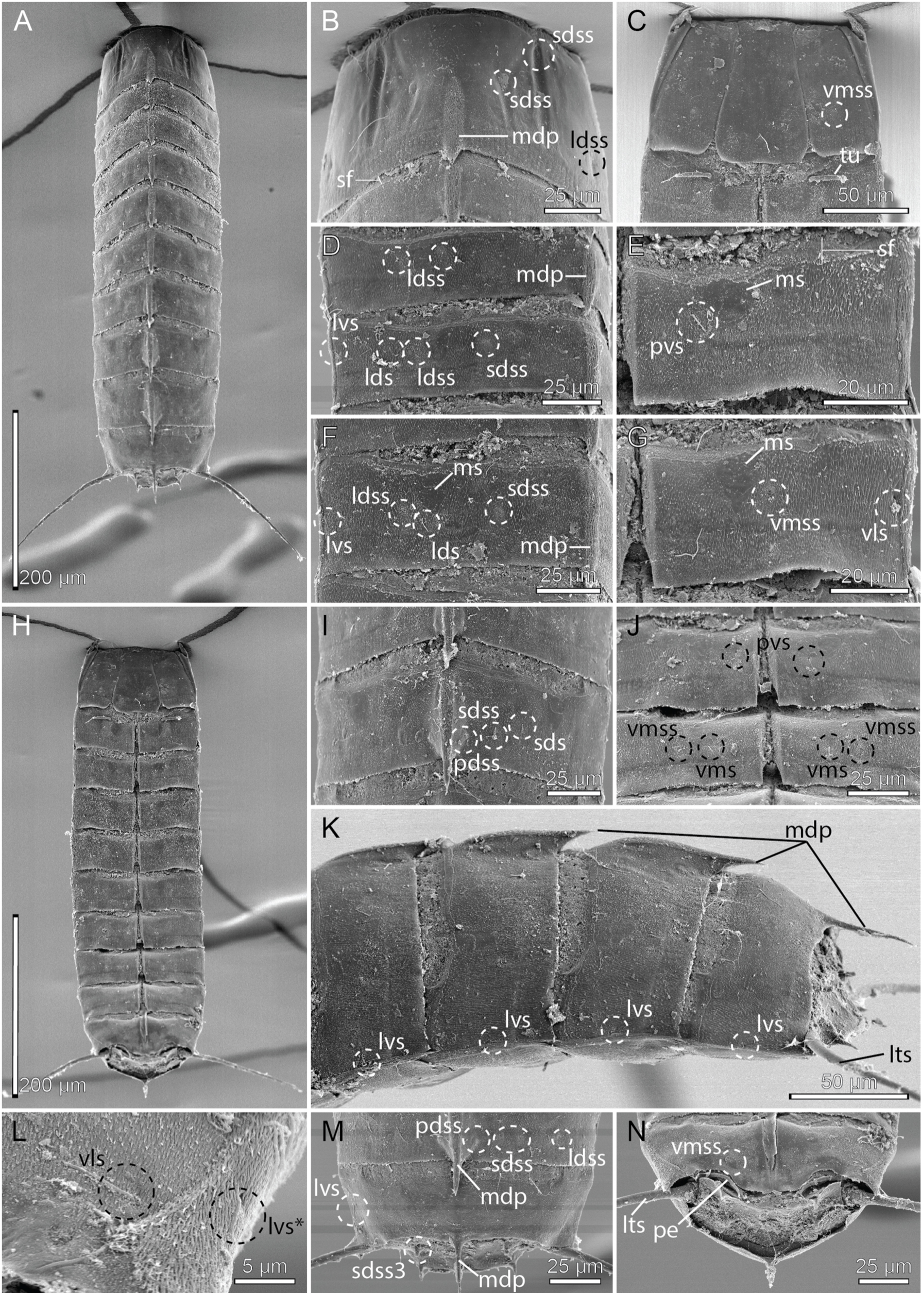
Scanning electron micrographs showing overviews and details of male *Cristaphyes dordaidelosensis* sp. nov. (A) Dorsal overview. (B) Segments 1–2, dorsal view. (C) Segments 1–2, ventral view. (D) Segments 2–3, left side laterodorsal view. (E) Segment 3, left side sternal plate. (F) Segment 6, left side laterodorsal view. (G) Segment 5, left side sternal plate. (H) Ventral overview. (I) Segment 8, dorsal view. (J) Ventromedial and midventral areas of segments 7–8. (K) Segments 7–11, lateral view. (L) Detail of segment 10 showing the lateroventral and ventrolateral setae. (M) Segments 10–11, dorsal view. (N) Segments 10–11, ventral view. Abbreviations: lds, laterodorsal seta; ldss, laterodorsal sensory spot; lts, lateral terminal spine; lvs, lateroventral seta, * in (L) marks the additional lateroventral seta on segment 10; mdp, middorsal process; ms, muscular scar; pdss, paradorsal sensory spot; pe, penile spines; pvs, paraventral seta; sds, subdorsal seta; sdss, subdorsal sensory spot; sdss3, subdorsal sensory spot type 3; sf, secondary fringes; tu, tube; vls, ventrolateral seta; vmss, ventromedial sensory spot.

Class Allomalorhagida Sørensen et al., 2015
Family Pycnophyidae Zelinka, 1896
Genus *Cristaphyes* Sánchez et al., 2016
***Cristaphyes dordaidelosensis*** sp. nov.
urn:lsid:zoobank.org:act:12FCA0B9-084A-4C66-841B-186FCC3AFCD0
Figures 2–4, Tables 2 and 3

### Diagnosis

*Cristaphyes* with middorsal processes on segments 1–10, with the process of segment 10 projecting well beyond the terminal segment. Setae present in: subdorsal positions of segment 8, laterodorsal positions of segments 3–7 and 9, lateroventral positions of segments 2–10 inclusive one additional set of lateroventral setae on segment 10, ventrolateral positions of segments 5 and 10, ventromedial positions on segment 9, and paraventral positions of segments 3, 7, and 9; position of ventral setae on segment 8 vary between paraventral and ventromedial. Males with ventromedial tubes on segment 2; female morphology unknown. Posterolateral processes of segment 10 acute. Lateral terminal spines present.

### Etymology

The species name *dordaidelosensis*, meaning “living in Dor Daidelos,” is inspired by the book Silmarillion by JRR Tolkien. According to the book, Dor Daidelos is “The Region of Everlasting Cold” and the northernmost region of Middle Earth in the First Age.

### Material examined

Holotype, adult male, collected from mud on August 2, 2013, on St. H6 at 236 m depth in Hornsund (70°40.98′N 014°48.73′E), mounted in Fluoromount G, deposited at the Natural History Museum of Denmark, under catalogue number NHMD-233049. Paratypic material includes one male from St. KB2, Kongsfjorden, mounted in Fluoromount G, and deposited at the Natural History Museum of Denmark, under catalogue number NHMD-233050. Additional non-type specimens include one male from St. A4, south of Nordaustlandet, mounted for SEM and stored in the first author’s personal reference collection. See Fig. 1 for localities and Table 1 for detailed station data.

### Description

Adults with head, neck, and eleven trunk segments (Figs. 2, 3A, 4A and 4H). The trunk is nearly parallel sided from segments 1 to 8. The terminal segment is almost completely covered by segment 10. Segment 1 consists of a tergal, two episternal and a midsternal plate (Figs. 2B, 3C and 4C), whereas the following ten segments consist of a tergal and two sternal plates. Lateral terminal spines are present, and about the same length as segments 8–10. Only male morphology is known. For complete overview of measures and dimensions, see Table 2. Distribution of cuticular structures, that is, sensory spots, tubes, and setae, is summarized in Table 3.

**Table 2:** Measurements from light microscopy of male holotype and paratype of *Cristaphyes dordaidelosensis* sp. nov. (in μm).

Character	Holotype	Paratype
TL	717	689
MSW-7	194	181
MSW-7/TL	27.1%	26.3%
SW-10	157	167
SW-10/TL	21.9%	24.2%
S1	117	113
S2	83	72
S3	84	77
S4	85	77
S5	86	79
S6	86	82
S7	87	83
S8	91	85
S9	88	78
S10	88	88
S11	39	45
MDP10	36	38
LTS	199	202
LTS/TL	27.8%	29.3%

**Note:**

LTS, lateral terminal spine; MDP10, middorsal process on segment 10; MSW-7, maximum sternal width, measured on segment 7 in this species; S, segment lengths; SW-10, standard width, always measured on segment 10; TL, trunk length.

**Table 3:** Summary of nature and location of sensory spots, setae, and tubes arranged by series in *Cristaphyes dordaidelosensis* sp. nov.

Position segment	PD	SD	LD	LV	VL	VM	PV
1	ss	ss, ss	ss			ss	
2	ss		ss, ss	se		tu(♂), ss	
3	ss	ss	ss, se	se			se
4	ss	ss	ss, se	se		ss	
5	ss	ss	ss, se	se	se	ss	
6	ss	ss	se, ss	se		ss	
7	ss	ss	se, ss	se		ss	se
8	ss	ss, se	ss	se		ss	se
9	ss	ss, ss	se, ss	se	ss	se	ss, se
10		ss, ss		se, se	se	ss	
11		ss3		lts	pe, pe(♂)		

**Note:**

LD, Laterodorsal; LV, lateroventral; PD, paradorsal; PV, paraventral; SD, subdorsal; VL, ventrolateral; VM, ventromedial; lts, lateral terminal spine; pe, penile spines; se, seta; ss, sensory spot, 3 marks type 3 sensory spot; tu, tube; (♂), male condition of putative sexually dimorphic character.

The head was fully retracted in the three examined specimens, hence no information on head morphology is available. The neck has four dorsal and two ventral placids; all placids are rectangular, and the most dorsal pair appears to be broadest, but exact measures of their width could not be obtained.

Middorsal processes are present on segments 1–10 (Fig. 2A); processes on segments 1–7 project only slightly beyond the posterior segment margins, but they become gradually longer at the more posterior segments (Fig. 4K). The strong middorsal processes of segments 8 and 9 project well beyond the posterior segment margins, and the relatively long but thinner middorsal process of segment 10 projects beyond the trunk (Figs. 2A, 3H–3I, 4K and 4M). Rounded to oval glandular cell outlets are present in series on the dorsal and ventral sides, in subdorsal positions on segment 1 and 8–10 (Figs. 2A, 3B and 3I), in laterodorsal positions in segments 2–7 (Figs. 2A, 3D and 3F), and in ventromedial positions on segments 1–10, located on the episternal plates, and hence more laterally displaced on segment 1 (Figs. 2B, 3C, 3E and 3G). Smooth, hairless areas (muscle scars) marking subcuticular muscle attachment sites are present anteriorly on the segments, in laterodorsal and ventromedial positions on segments 2–9 (Figs. 2, 4E and 4F). Segment 1 is basically smooth, whereas segments 2–10 are covered with very minute acicular hairs. Secondary fringes, formed by one to two bands, are present on segments 2–10. Pachycycli, and rounded to oval peg-and-socket joints are present on segments 2–10 (Fig. 4B). Paraventral apodemes are absent on all segments.

Segment 1 with middorsal process, rising medially on segment, and projecting slightly beyond the posterior segment margin; ridge of process is covered by densely set hairs (Fig. 4B). Midsternal plate trapezoid, but with lateral sides close to being parallel (Figs. 3C and 4C). Anterior segment margin with narrow reticulated area along the margins of the tergal and midsternal plates, and larger reticulated areas at the anteroventral corners of the episternal plates. The segmental plates terminate posteriorly in free flaps, with finely serrated margins. Sensory spots present in paradorsal positions at posterior segment margin near projecting part of middorsal process; and as two pairs in subdorsal positions, one pair medially on segment and the other more anterior (Figs. 2A and 4B); both pairs appear to be located in the anterior ends of elongated depressed areas in the cuticle. Sensory spots furthermore present in laterodorsal (Fig. 4B) and ventromedial (Fig. 4C) positions, more posteriorly on segment.

Segment 2 with middorsal process and paradorsal sensory spots as on preceding segment, but with ridge of middorsal process expanding from the most anterior part of the segment (Figs. 2A, 4B and 4D). Tergal plate furthermore with two pairs of laterodorsal sensory spots (Fig. 4D), flanking the muscle scar, and a pair of lateroventral setae. Sternal plates with ventromedial tubes (Figs. 2B, 3C and 4C), putatively representing a sexually dimorphic male character, and ventromedial sensory spots, located posterior to the tubes (Fig. 3C). Posterior segment margin as on preceding segment.

Segment 3 with middorsal process and paradorsal sensory spots as on preceding segment (Figs. 2A, 3D and 4D). Tergal plate furthermore with subdorsal and laterodorsal sensory spots, and laterodorsal and lateroventral setae; laterodorsal setae are located more lateral than the sensory spots in same position (Fig. 4D). Sternal plates with paraventral setae (Figs. 2B, 3E and 4E).

Segment 4 with middorsal process being slightly longer than on preceding segment and paradorsal sensory spots. Tergal plate otherwise as segment 3 (Figs. 2A and 3D). Sternal plates with ventromedial sensory spots (Fig. 2B).

Segment 5 with tergal plate as on preceding segment, but with slightly longer middorsal process (Figs. 2A and 3D). Sternal plates with ventrolateral setae and ventromedial sensory spots (Figs. 2B, 3E and 4G).

Segment 6 with tergal plate as on preceding segment, but with slightly longer middorsal process, and with laterodorsal sensory spots and setae in switched positions, so that the sensory spots now are more lateral (Figs. 2A, 3F and 4F). Sternal plates as on segment 4 (Figs. 2B).

Segment 7 with tergal plate as on segment 6, but with slightly longer middorsal process (Figs. 2A, 3F and 4K). Sternal plates with ventromedial sensory spots and paraventral setae (Figs. 2B, 3G and 4J).

Segment 8 with longer middorsal process, flanked by paradorsal sensory spots; sensory spots are not as close to the posterior segment margin as those on preceding segments. Tergal plate otherwise with subdorsal sensory spots (located slightly closer to the middorsal process than those on preceding segments), subdorsal setae, laterodorsal sensory spots, and lateroventral setae (Figs. 2A, 3I, 4I and 4K). Sternal plates with setae in paraventral areas in one specimen (Fig. 3G), or in ventromedial positions, but very close to the paraventral areas in another specimen (Fig. 4J). Sensory spots present more lateral, also in ventrolateral positions (Figs. 2B and 4J).

Segment 9 with even longer middorsal process, flanked by paradorsal sensory spots as on segment 8. Tergal plate otherwise with two pairs of subdorsal sensory spots, laterodorsal setae, laterodorsal sensory spots, and lateroventral setae (Figs. 2A, 3I, 4K and 4M). Sternal plates with ventrolateral and paraventral sensory spots, and ventromedial and paraventral setae, with the latter pair being located very close to the midsternal junction (Figs. 2B and 3G).

Segment 10 with middorsal process without conspicuous middorsal ridge, expanding from the posterior segment margin, and projecting well beyond the terminal end of the trunk (Figs. 2A, 3H, 4K and 4M). Tergal plate otherwise with two pairs of sensory spots in subdorsal positions (near posterior segment margin), and two pairs of setae in lateroventral positions; the dorsalmost pair is perfectly aligned with the lateroventral setae on the preceding segments, whereas the additional pair is more ventral, and appears to attach directed in the densely haired area near the tergosternal junction (Fig. 4L). Sternal plates with ventrolateral setae and ventromedial sensory spots, both near posterior segment margin (Figs. 2B, 3J, 4L and 4N). Posterior margin of tergal plate is straight as on all preceding segments, whereas the margins of the sternal plates are concave (Figs. 2B, 3J and 4N). Posterolateral processes of tergosternal junctions form pointed, acute projections (Fig. 3J).

Segment 11 hardly projecting beyond segment 10. Lateral terminal spines present (Figs. 2, 3A, 4A and 4N). Tergal plate with pair of slightly projecting type 3 sensory spots in subdorsal positions (Figs. 3H and 4M). Margin of sternal plates with pair of pointed, horn-like projections (Fig. 3J). Two pairs of penile spines present (Figs. 2B, 3J and 4N).

### Remarks for *Cristaphyes dordaidelosensis* sp. nov.

Of the additional 19 species accommodated in *Cristaphyes*, *C. dordaidelosensis* sp. nov. is easily distinguished from the eight species that do not have lateral terminal spines. Of the eleven remaining species, five of them have none or only very short middorsal process of segment 10 that does not project beyond the posterior margin of the segment. These include *C. carinatus* (Zelinka, 1928), *C. chilensis* (Lang, 1953), *C. cryopygus*, *C. longicornis* (Higgins, 1983), and *C. odhneri* (Lang, 1949). *C. dordaidelosensis* sp. nov. is easily distinguished from these by its long projecting middorsal process of segment 10 (see Zelinka, 1928; Lang, 1949, 1953; Higgins, 1983; Higgins & Kristensen, 1988).

The six remaining species with lateral terminal spines, and conspicuously projecting middorsal process of segment 10 include: *C. arctous*, *C. chukchiensis*, *C. cristatus* (Sánchez et al., 2013), *C. furugelmi* (Adrianov, 1999 in Adrianov & Malakhov, 1999), *C. nubilis* (Sánchez, Pardos & Sørensen, 2014), and *C. abyssorum* (Adrianov & Maiorova, 2015) (see Higgins, 1991; Adrianov & Malakhov, 1999; Sánchez et al., 2013; Sánchez, Pardos & Sørensen, 2014; Adrianov & Maiorova, 2015). *C. dordaidelosensis* sp. nov. is most easily distinguished from these by the distribution of setae on its sternal plates. *C. dordaidelosensis* sp. nov. has relatively few ventral setae, and besides the ventrolateral pair on segment five that is present in all *Cristaphyes* species, it mostly has paraventral setae on segments 3, 7, and 9, and occasionally 8. Oppositely, *C. abyssorum*, *C. chukchiensis*, and *C. nubilis* have ventromedial setae on a majority of their segments. *C. abyssorum* has ventromedial setae on segments 1, plus 3–9 (Adrianov & Maiorova, 2015), and *C. chukchiensis* and *C. nubilis* in ventromedial positions on segments 2–9 (Higgins, 1991; Sánchez, Pardos & Sørensen, 2014). *C. furugelmi* has fewer ventral setae, but it has ventromedial setae on segments 4–6 and 10 (Adrianov & Malakhov, 1999), which are segments where *C. dordaidelosensis* sp. nov. has neither paraventral nor ventromedial setae. *C. arctous* is also easily distinguished from *C. dordaidelosensis* sp. nov. by its apparent lack of ventral setae in general (Adrianov & Malakhov, 1999), but an even more conspicuous, differential character is its middorsal processes that are shorter and more obtuse on segments 1–8 than those in *C. dordaidelosensis* sp. nov. Oppositely, *C. dordaidelosensis* sp. nov. has more pointed and projecting middorsal processes on all segments, from segment 1 to 10. *C. cristatus* show some resemblance with *C. dordaidelosensis* sp. nov. Both species have ventral setae on segments 7–9, but in addition *C. dordaidelosensis* sp. nov. has paraventral setae on segment 3, and two pairs of setae (ventromedial and paraventral) on segment 9 and three pairs on segment 10. The two species furthermore differ considerably on their tergal plates, where *C. cristatus* has no subdorsal or laterodorsal setae at all from segment 2 to 6 (Sánchez et al., 2013), and lateroventral setae on even numbered segments only. Oppositely, *C. dordaidelosensis* sp. nov. has laterodorsal setae on segments 3–7 and on 9, and lateroventral setae on all segments from segment 2 to 10.

In summary, *C. dordaidelosensis* sp. nov. is distinguished by its combination of lateral terminal spines, middorsal processes on segments 1–10, with the process on segment 10 clearly projecting beyond the terminal segment, and by its relatively few ventral setae, mostly in paraventral positions.

**Figure 5:**
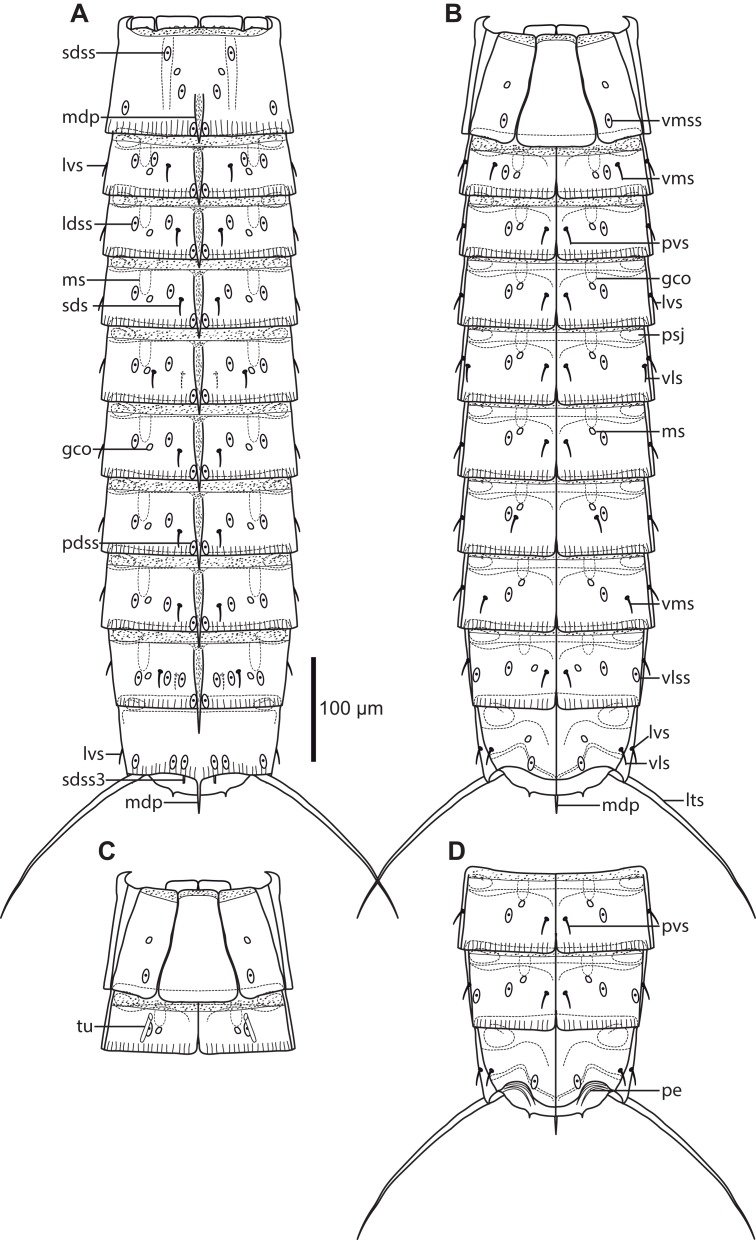
Line art illustrations of *Cristaphyes glaurung* sp. nov. (A) Female, dorsal view. (B) Female, ventral view. (C) Male, segments 1–2, ventral view. (D) Male, segments 8–11, ventral view. Abbreviations: gco, glandular cell outlet; ldss, laterodorsal sensory spot; lts, lateral terminal spine; lvs, lateroventral seta; mdp, middorsal process; ms, muscular scar; pdss, paradorsal sensory spot; pe, penile spines; pvs, paraventral seta; sds, subdorsal seta; sdss, subdorsal sensory spot; sdss3, subdorsal sensory spot type 3; psj, peg-and-socket joint; tu, tube; vls, ventrolateral seta; vlss, ventrolateral sensory spot; vms, ventromedial seta; vmss, ventromedial sensory spot. Setae drawn with dashed lines indicate alternative position of setae showing intraspecific variation.

**Figure 6:**
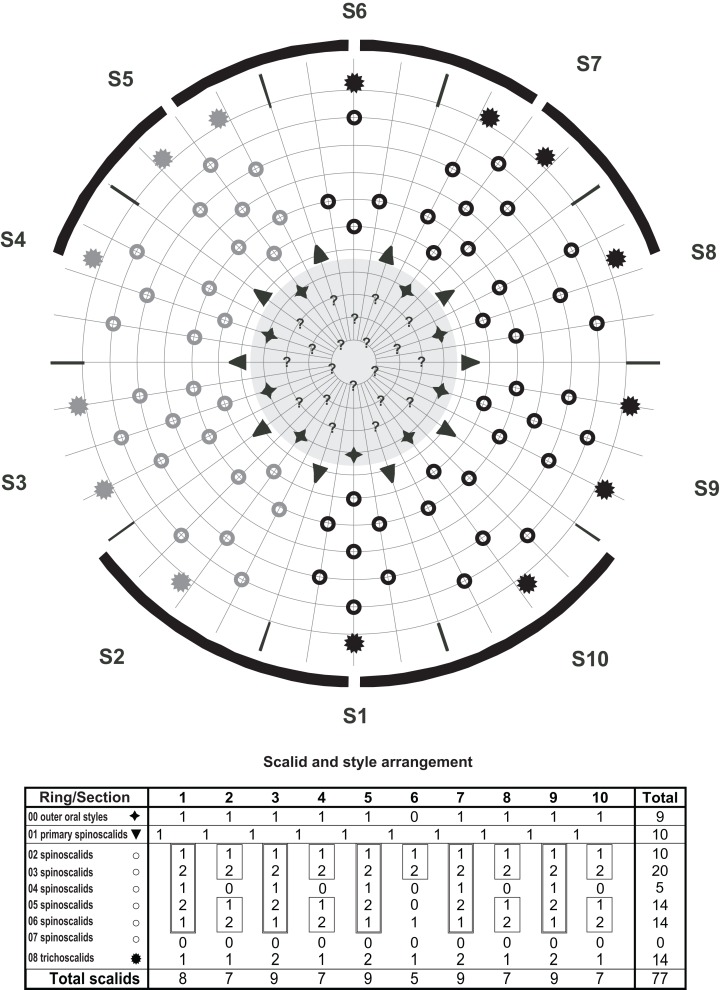
Diagram of mouth cone (gray area), introvert, and placids in *Cristaphyes glaurung* sp. nov., showing distribution of outer oral styles, scalids, and trichoscalids. Scalids in gray have not been visually confirmed, but are marked based on the assumption that the introvert is bilateral symmetrical. Table shows the scalid arrangement by sector; single-lined boxes mark chevrons, double-lined boxes mark “double diamonds.”

**Figure 7:**
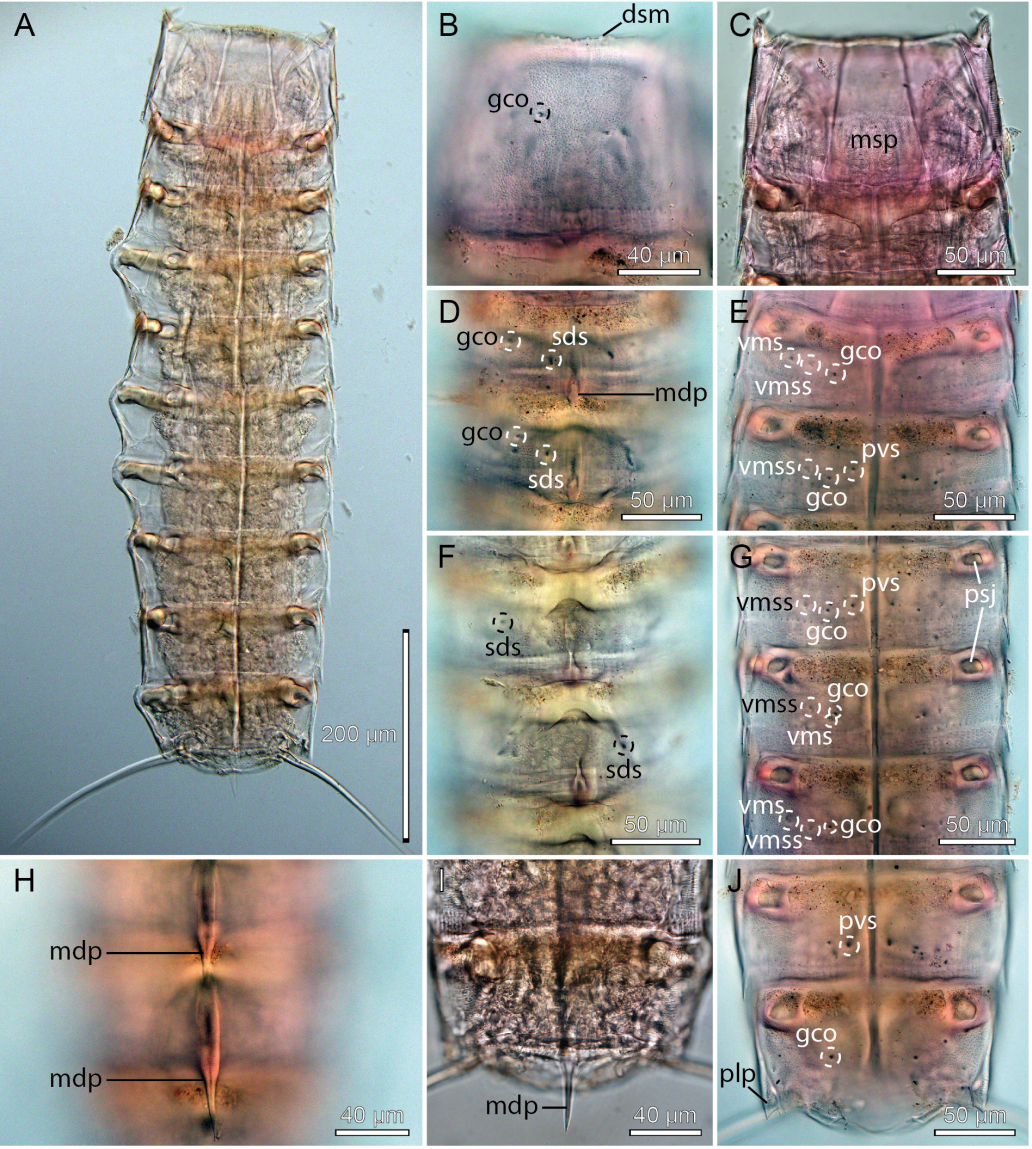
Light micrographs showing overviews and details of female holotype, NHMD-233053, of *Cristaphyes glaurung* sp. nov. (A) Ventral overview. (B) Segment 1, dorsal view. (C) Segments 1–2, ventral view. (D) Segments 3–4, dorsal view. (E) Segments 2–3, ventral view. (F) Segments 5–6, dorsal view. (G) Segments 6–8, ventral view. (H) Segments 8–9, focused at middorsal processes. (I) Segments 9–10, focused at middorsal process. (J) Segments 9–11, ventral view. Abbreviations: dsm, denticulated segment margin; gco, glandular cell outlet; mdp, middorsal process; msp, midsternal plate; plp, posterolateral process; psj, peg-and-socket joint; pvs, paraventral seta; sds, subdorsal seta; vms, ventromedial seta; vmss, ventromedial sensory spot.

**Figure 8:**
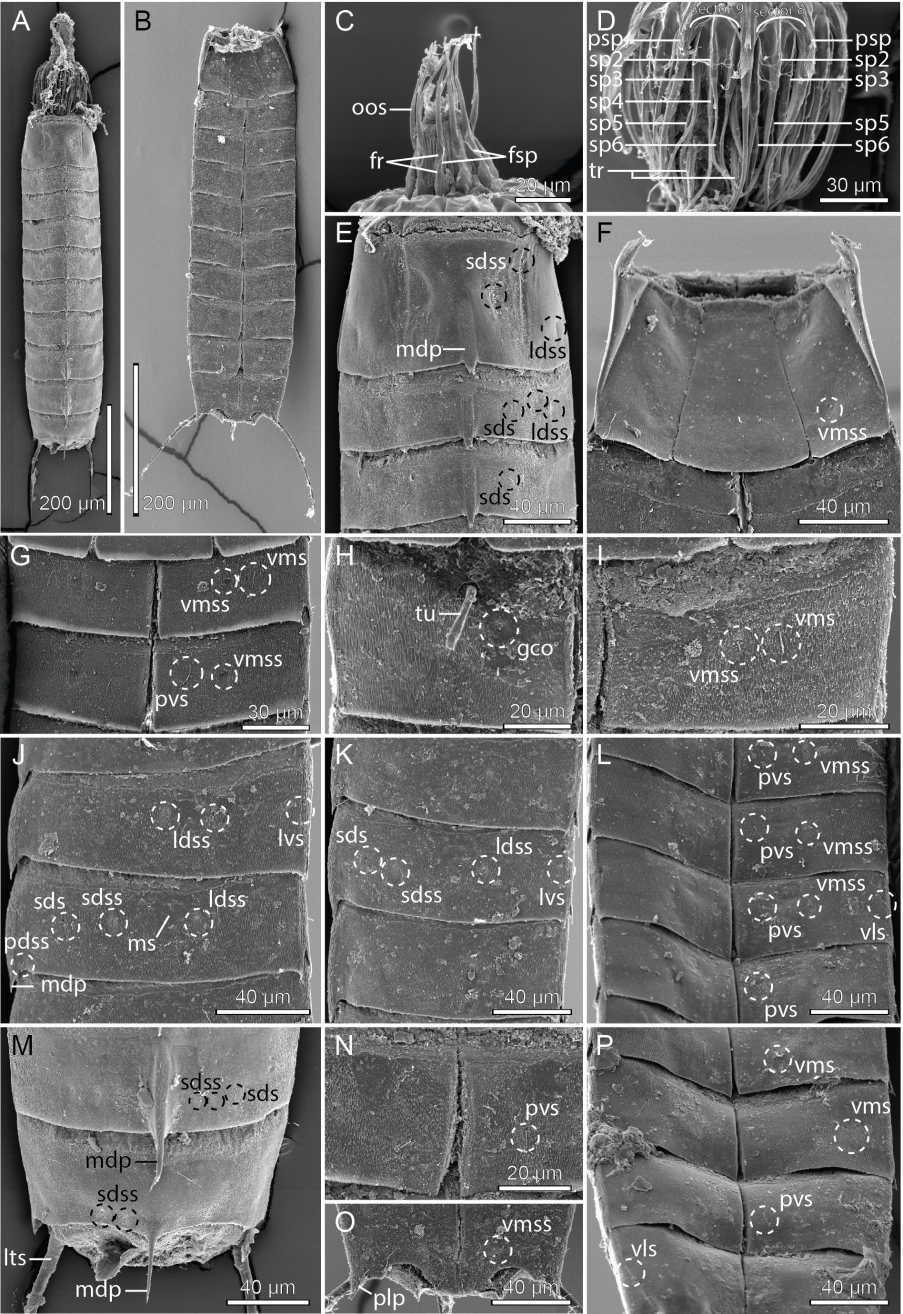
Scanning electron micrographs showing overviews and details of *Cristaphyes glaurung* sp. nov. (A) Dorsal overview of female. (B) Ventral overview of male. (C) Detail of head showing mouth cone with outer oral styles. (D) Detail of head showing introvert sectors 9 and 8. (E) Segments 1–3, dorsal view. (F) Segment 1, ventral view. (G) Segments 2 and 3, ventral view in female. (H) Detail showing segment 2, right sternal pate in male. (I) Detail showing segment 2, left sternal pate in female. (J) Segment 2–3, right side tergal plates. (K) Segment 5–7, right side tergal plates. (L) Segments 3–6, ventral view. (M) Segments 9–11, dorsal view of female. (N) Segment 8, sternal plates in male. (O) Segments 10–11, ventral view of male. (P) Segments 7–10, ventral view of female. Abbreviations: fr, fringe; fsp, fringe spike; gco, glandular cell outlet; ldss, laterodorsal sensory spot; lts, lateral terminal spine; lvs, lateroventral seta; mdp, middorsal process; ms, muscular scar; oos, outer oral styles; pdss, paradorsal sensory spot; plp, posterolateral process; psp, primary spinoscalid; pvs, paraventral seta; sds, subdorsal seta; sdss, subdorsal sensory spot; sp, spinoscalid followed by introvert ring number; tr, trichoscalid; tu, tube; vls, ventrolateral seta; vms, ventromedial seta; vmss, ventromedial sensory spot.

***Cristaphyes glaurung*** sp. nov.
urn:lsid:zoobank.org:act:7955F387-C093-4823-A416-58A9D2734833
Figures 5–8, Tables 4 and 5

### Diagnosis

*Cristaphyes* with middorsal processes on segments 1–10, with the process of segment 10 projecting well beyond the terminal segment. Setae present in: subdorsal positions of segments 2–9 (setae on segments 5 and 9 may vary from sub- to laterodorsal positions), lateroventral positions of segments 2–10, and ventrolateral positions of segments 5 and 10; females furthermore with setae in ventromedial positions on segments 2, 7, and 8 and in paraventral positions on segments 3–6, and 9; males with ventromedial tubes on segment 2, and setae in paraventral positions in segments 3–9. Posterolateral processes of segment 10 form nearly right angle. Lateral terminal spines present.

### Etymology

The species is named *glaurung*, after Glaurung—the father of the dragons, bread by Morgoth in the dungeons of Angband—in the book Silmarillion by JRR Tolkien.

### Material examined

Holotype, adult female, collected from mud on May 24, 2016, on St. A4 at 217 m depth south of Nordaustlandet (79°12.53′N 025°59.74′E), mounted in Fluoromount G, deposited at the Natural History Museum of Denmark, under catalogue number NHMD-233053. Paratypes include one female from same locality as the holotype, one male from St. A1 in Van Mijenfjorden, one female from St. A2 in Hornsund, one female and one male from St. KB2 in Kongsfjorden (all three fjords on the west coast of Spitsbergen), and one female from St. A3 in Storfjorden between Spitsbergen and Edgeøya, all mounted in Fluoromount G, and deposited at the Natural History Museum of Denmark, under catalogue numbers NHMD-233054–233059. Additional non-type specimens include three females and one male from St. A3, one female from St. A4, and two females and one male from St. KG1 in Kongsfjorden, mounted for SEM and stored in the first author’s personal reference collection. See Fig. 1 for localities and Table 1 for detailed station data.

### Description

Adults with head, neck and eleven trunk segments (Figs. 5A–5B, 6, 7A and 8A–8B). The trunk is nearly parallel sided from segments 1 to 9. The terminal segment is almost completely covered by segment 10. Segment 1 consists of a tergal, two episternal and a midsternal plate (Figs. 5B, 7C, 8F), whereas the following ten segments consist of a tergal and two sternal plates. Lateral terminal spines are present, and about the same length as segments 8–10. For complete overview of measures and dimensions, see Table 4. Distribution of cuticular structures, that is, sensory spots, tubes and setae, is summarized in Table 5.

**Table 4:** Measurements from light microscopy of *Cristaphyes glaurung* sp. nov. (in μm), including number of measured specimens (*n*) and standard deviation (SD).

Character	*n*	Range	Mean	SD
TL	7	685–763	729	24.31
MSW-7	7	158–173	165	5.22
MSW-7/TL	7	22.0–24.0%	22.7%	0.81%
SW-10	7	141–146	148	5.89
SW-10/TL	7	19.2–21.2%	20.3%	1.02%
S1	7	108–114	110	2.24
S2	7	66–74	70	3.64
S3	7	64–74	69	3.13
S4	7	66–80	72	4.86
S5	7	70–80	75	3.69
S6	7	70–82	77	3.99
S7	7	68–83	78	4.72
S8	7	77–84	81	2.61
S9	7	77–85	82	2.43
S10	7	82–84	83	0.90
S11	7	39–45	43	2.41
MDP10	7	39–45	41	2.29
LTS	7	190–203	194	5.16
LTS/TL	7	25.0–29.2%	26.7%	1.30%

**Note:**

LTS, lateral terminal spine; MDP10, middorsal process on segment 10; MSW-7, maximum sternal width, measured on segment 7 in this species; S, segment lengths; SW-10, standard width, always measured on segment 10; TL, trunk length.

**Table 5:** Summary of nature and location of sensory spots, setae, and tubes arranged by series in *Cristaphyes glaurung* sp. nov.

Position segment	PD	SD	LD	LV	VL	VM	PV
1	ss	ss, ss	ss			ss	
2	ss	se	ss, ss	se		tu(♂), se(♀), ss	
3	ss	se, ss	ss	se		ss	se
4	ss	se, ss	ss	se		ss	se
5	ss	ss	se*, ss	se	se	ss	se
6	ss	se, ss	ss	se		ss	se
7	ss	se, ss	ss	se		ss, se(♀)	se(♂)
8	ss	se, ss	ss	se		ss, se(♀)	se(♂)
9	ss	ss, ss, se^¤^	ss	se	ss	ss	se
10		ss, ss	ss	se	se	ss	
11		ss3		lts	pe, pe(♂)		

**Notes:**

LD, Laterodorsal; LV, lateroventral; PD, paradorsal; PV, paraventral; SD, subdorsal; VL, ventrolateral; VM, ventromedial; lts, lateral terminal spine; pe, penile spines; se, seta; ss, sensory spot, 3 marks type 3 sensory spot; tu, tube; (♂) male and (♀) female conditions of sexually dimorphic characters.

* Marks setae that differ in position from nearly paradorsal to laterodorsal, but most commonly occur in laterodorsal positions.

¤ Marks setae missing in some specimens.

A single specimen mounted for SEM had its head fully everted, enabling visual examination of introvert sectors 1–2, and 6–10. Hence, a full description of the introvert can be provided (Fig. 6), assuming that the introvert shows the usual symmetry patterns, and that introvert sectors 3–5 are identical with sectors 7–9, respectively. The mouth cone has nine outer oral styles (Fig. 8C), arranged as one anterior to each introvert sector, expect for the middorsal sector 6 (Fig. 6). Each outer oral style consists of a single, rather flexible unit (Fig. 8C). Proximally they attach to the mouth cone via a fringed sheath. An additional fringed structure is present externally on this sheath, and every second of these structures (anterior to even-numbered introvert sectors) carry a long, spike-like median fringe tips (Fig. 8C).

The introvert is equipped with spinoscalids, arranged in transverse rings and longitudinally in ten sectors, defined by the primary spinoscalids of ring 01 (Fig. 6). The primary spinoscalids consist of a stout proximal unit with a median fringe, and a long, slender end piece (Fig. 8D). Spinoscalids of rings 02 and 03 have more narrow proximal sheaths, with rather long median fringes. End pieces are long, and more slender than those in ring 01. Spinoscalids of the remaining rings also consist of a proximal sheath and an end piece, but they are shorter, and the conspicuous median fringe is missing. Instead, they appear to have a smaller, transverse fringe near the attachment points. Ring-wise arrangement of spinoscalids is as follows: Ring 01—10 primary spinoscalids; Ring 02—10 spinoscalids, one medially in each sector; Ring 03—20 spinoscalids, one pair in each sector; Ring 04—five spinoscalids, one medially but in uneven-numbered sectors only; Ring 05—14 spinoscalids, one pair in uneven-numbered sectors, and one medially in even-numbered sectors, except the middorsal sector 6; Ring 06—14 spinoscalids, one medially in uneven-numbered sectors and sector 6, and one pair in remaining sectors (Figs. 6 and 8D). Described sector-wise, all uneven-numbered sectors have seven spinoscalids, arranged as a double diamond. Even-numbered sectors, except sector 6, have spinoscalids forming two chevrons (i.e., a single spinoscalid anterior to a pair in each chevron), with a blank ring separating the two chevrons. Sector 6 has a chevron in Rings 02–03, and then a single, medial spinoscalid in Ring 06. A total of 14 trichoscalids are present posterior to the spinoscalid rings. They are located as single trichoscalids in even-numbered sectors, and in sector 1, and as pairs in the remaining uneven-numbered sectors (Fig. 6).

The neck has four dorsal and two ventral placids; all placids are rectangular and measures in width: 33 μm (subdorsal pair) and 26 μm (laterodorsal and ventral pairs), respectively.

Middorsal processes are present on segments 1–10; processes on segments 1–7 project only slightly beyond the posterior segment margins, but they become gradually longer at the more posterior segments (Figs. 5A, 7D, 7H, 8A, 8E, 8J and 8M). The strong middorsal processes of segments 8 and 9 project well beyond the posterior segment margins, and the relatively long but thinner middorsal process of segment 10 projects beyond the trunk. Rounded to oval glandular cell outlets are present in series on the dorsal and ventral sides (Figs. 5A–5B, 7B, 7D–7E, 7G and 7J), in subdorsal positions on segment 1, in laterodorsal positions in segments 2–9, and in ventromedial positions on segments 1–10. Smooth, hairless areas (muscle scars) marking subcuticular muscle attachment sites are present anteriorly on the segments (Figs. 5A and 5B), in laterodorsal and ventromedial positions on segments 2–9. All segments, except anterior and lateral parts of segment 1, are covered with very minute acicular hairs. Secondary fringes, formed by one to two wavy bands, are present in segments 2–10. Pachycycli, and peg-and-socket joints are present on segments 2–10. Paraventral apodemes are absent on all segments.

Segment 1 with middorsal process, rising on posterior 1/3 of segment, and projecting slightly beyond the posterior segment margin; ridge of process is covered by densely set hairs (Figs. 5A and 8E). Midsternal plate Erlenmeyer flask-shaped (Figs. 7C and 8F). Anterior segment margin denticulated (Fig. 7B) with narrow reticulated area along the margins of the tergal and midsternal plates, and slightly larger reticulated areas along margins of episternal plates (Figs. 5A–5C). The segmental plates terminate posteriorly in free flaps, with finely serrated margins. Sensory spots present in paradorsal positions at posterior segment margin near projecting part of middorsal process; and as two pairs in subdorsal positions, one pair medially on segment and the other more anterior; anterior pair appears to be located in the anterior ends of elongated depressed areas in the cuticle (Figs. 5A and 8E). Sensory spots furthermore present in laterodorsal (Fig. 8E) and ventromedial (Figs. 5B and 8F) positions, more posteriorly on segment.

Segment 2 with middorsal process and paradorsal sensory spots as on preceding segment, but with ridge of middorsal process expanding from the most anterior part of the segment (Fig. 8E). Tergal plate furthermore with two pairs of laterodorsal sensory spots, flanking the muscle scar, and a pair of subdorsal and lateroventral setae (Figs. 5A, 7E, 8E and 8J). Sternal plates with ventromedial sensory spots in both sexes located lateral to glandular cell outlets; females furthermore with ventromedial setae, located lateral to the sensory spots (Figs. 5B, 7E, 8G and 8I), and males with ventromedial tubes (Figs. 5C and 8H). Posterior segment margin as on preceding segment.

Segment 3 with middorsal process and paradorsal sensory spots as on preceding segment. Tergal plate furthermore with subdorsal and laterodorsal sensory spots, and subdorsal and lateroventral setae; subdorsal setae are located more dorsal than those on segment 2 (Figs. 5A, 7D and 8E). Sternal plates with ventromedial sensory spots and paraventral setae (Figs. 5B, 7E, 8G, 8J and 8L).

Segment 4 similar to preceding segment (Figs. 5A–5B, 7D and 8L).

Segment 5 with tergal plate as on preceding segment, except for the slightly longer middorsal process, and setae that vary in positions between subdorsal and laterodorsal, but most commonly occur in laterodorsal positions (Figs. 5A, 7F and 8K); the variation appears to be due to random intraspecific variation rather than sexual dimorphism. Sternal plates with ventrolateral and paraventral setae and ventromedial sensory spots (Figs. 5B and 8L).

Segment 6 with slightly longer middorsal process, but otherwise as segment 4 (Figs. 5A–5B, 7F–7G and 8K–8L).

Segment 7 with tergal plate as on preceding segment, but with slightly longer middorsal process (Figs. 5A and 8K). Sternal plates with ventromedial sensory spots in both sexes; females furthermore with setae in ventromedial positions (Figs. 7G and 8P), and males with setae in paraventral positions.

Segment 8 with tergal plate as on preceding segment, but with longer middorsal process (Figs. 5A and 7H). Sternal plates as segment 7, displaying the same sexual dimorphism (Figs. 5B, 5D, 7G, 8N and 8P), but with the ventromedial setae in females located even more lateral (Fig. 8P).

Segment 9 with even longer middorsal process, flanked by paradorsal sensory spots. Tergal plate otherwise with two pairs of subdorsal sensory spots, mostly subdorsal setae (but varying in position from nearly paradorsal to nearly laterodorsal), laterodorsal sensory spots, and lateroventral setae (Figs. 5A, 7H and 8M). Sternal plates with ventrolateral and ventromedial sensory spots, and paraventral setae in both sexes (Figs. 5B, 5D, 7J and 8P).

Segment 10 with middorsal process without conspicuous middorsal ridge and flanking sensory spots, expanding from the posterior segment margin, and projecting well beyond the terminal end of the trunk (Figs. 5A, 7I and 8M). Tergal plate otherwise with two pairs of sensory spots in subdorsal and one pair in laterodorsal positions (all near posterior segment margin), and pair of lateroventral setae. Sternal plates with ventrolateral setae and ventromedial sensory spots, near posterior segment margin (Figs. 5B, 5D, 7J and 8O–8P). Posterior margin of tergal plate is straight as on all preceding segment, whereas the margins of the sternal plates are deeply concave in males (Figs. 5D and 8O), but only slightly concave in females (Fig. 5B). Posterolateral corners of tergosternal junctions form nearly right-angled caudal projections (Figs. 7J and 8O).

Segment 11 hardly projecting beyond segment 10. Lateral terminal spines present (Figs. 5A–5B, 7A and 8M). Tergal plate with pair of slightly projecting type 3 sensory spots in subdorsal positions. Margin of sternal plates with pair of pointed, horn-like projections. Two pairs of penile spines present in males (Fig. 5D).

### Remarks for *Cristaphyes glaurung* sp. nov.

*Cristaphyes glaurung* sp. nov. is also easily distinguished from the eight congeners without lateral terminal spines, and the five additional ones with no or only very short middorsal process of segment 10. The remaining species with lateral terminal spines, and slightly or conspicuously projecting middorsal process of segment 10 include: *C. arctous*, *C. chukchiensis*, *C. cristatus*, *C. furugelmi*, *C. nubilis*, *C. abyssorum*, and the species described above, *C. dordaidelosensis* sp. nov. *C. glaurung* sp. nov. is fairly easily distinguished from these by its paraventral setae on segments 3–9 in males, and 3–6 plus 9 in females. *C. abyssorum* and *C. nubilis* also have sternal setae on most segments, but they are never paraventral, and their ventromedial setae are quite lateral (Adrianov & Maiorova, 2015; Sánchez, Pardos & Sørensen, 2014). Males of *C. furugelmi* have ventral setae on segment 7 that get close to the paraventral positions, but on its other segments, and in females, it has either quite laterally displaced ventromedial setae, or no sternal setae at all (Adrianov & Malakhov, 1999). As pointed out above, *C. arctous* apparently lacks sternal setae completely, and has conspicuously short and obtuse middorsal processes on segments 1–8 (Adrianov & Malakhov, 1999). Oppositely, *C. glaurung* sp. nov. has spinose and projecting middorsal processes on all segments, from segment 1 to 10. Also *C. cristatus* is easily distinguished from *C. glaurung* sp. nov. due to its lack sternal setae on segments 3 to 6 (Sánchez et al., 2013). In terms of seta distribution patterns, *C. chukchiensis* is closest to *C. glaurung* sp. nov. It has lateroventral setae on segments 2–10 (same in *C. glaurung* sp. nov.), laterodorsal setae on segments 2–9 (same segment distribution in *C. glaurung* sp. nov., but mostly in subdorsal positions), paraventral setae on segment 5 (same in *C. glaurung* sp. nov.), and ventromedial setae on segments 2–9 (same segment distribution in *C. glaurung* sp. nov., but mostly in paraventral positions) (Higgins, 1991). The two species can be distinguished by the subtle differences in positions of setae, but two better differential characters are the lack of a middorsal process on segment 1 in *C. chukchiensis*, and its broad trapezoid midsternal plate of segment 1 that differs from the Erlenmeyer flask-shaped midsternal plate in *C. glaurung* sp. nov. *C. dordaidelosensis* sp. nov. clearly differs from *C. glaurung* sp. nov. by its lack of sternal setae on segments 4 to 6. An even easier differential character, that was used during the initial identification of the species from Svalbard, is the shape of the posterolateral processes of segment 10. In *C. dordaidelosensis* sp. nov. the process is pointed and shaped by sides that meet in an acute angle, whereas the processes in *C. glaurung* sp. nov. have sides that forms a nearly right angle (compare processes shown on Fig. 3J with Fig. 7J). After seeing these differently shaped processes, it was very easy, even at low magnification, to distinguish the two species.

In summary, *C. glaurung* sp. nov. is distinguished by its combination of lateral terminal spines, middorsal processes on segments 1–10, with the process on segment 10 clearly projecting beyond the terminal segment, and by its ventral setae in paraventral positions, at least on segments 3–6 and 9.

The arrangement of spinoscalids on the introvert is identical with the pattern in *Pycnophyes chalgap* (Sánchez et al., 2013) and *C. cristatus* (see Sánchez et al., 2013).

### *Cristaphyes scatha* sp. nov.

**Figure 9:**
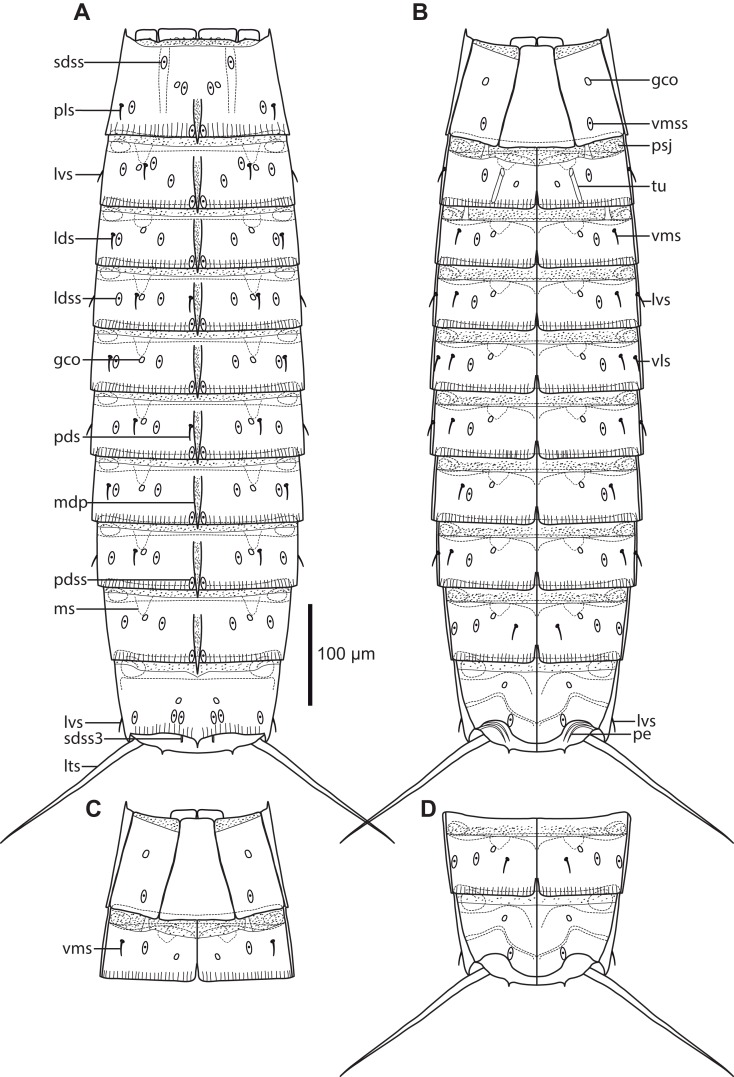
Line art illustrations of *Cristaphyes scatha* sp. nov. (A) Male, dorsal view. (B) Male, ventral view. (C) Female, segments 1–2, ventral view. (D) Female, segments 9–11, ventral view. Abbreviations: gco, glandular cell outlet; lds, laterodorsal seta; ldss, laterodorsal sensory spot; lts, lateral terminal spine; lvs, lateroventral seta; mdp, middorsal process; ms, muscular scar; pds, paradorsal seta; pdss, paradorsal sensory spot; pe, penile spines; pls, paralateral seta; psj, peg-and-socket joint; sdss, subdorsal sensory spot; sdss3, subdorsal sensory spot type 3; tu, tube; vls, ventrolateral seta; vms, ventromedial seta; vmss, ventromedial sensory spot.

**Figure 10:**
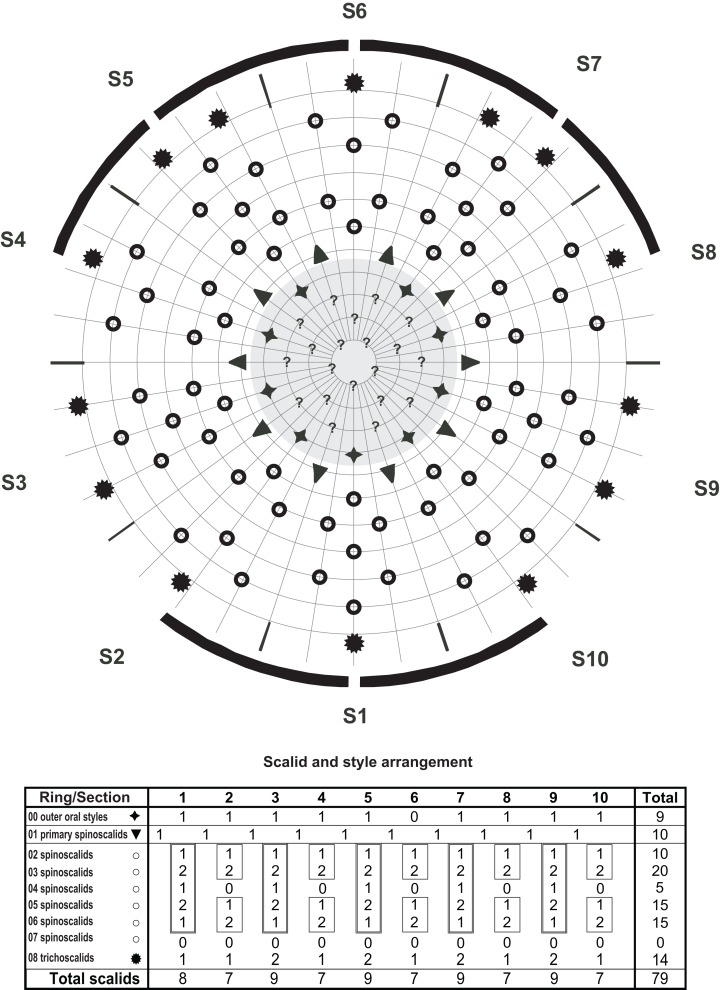
Diagram of mouth cone (gray area), introvert, and placids in *Cristaphyes scatha* sp. nov., showing distribution of outer oral styles, scalids, and trichoscalids. Table shows the scalid arrangement by sector; single-lined boxes mark chevrons, double-lined boxes mark “double diamonds.”

**Figure 11:**
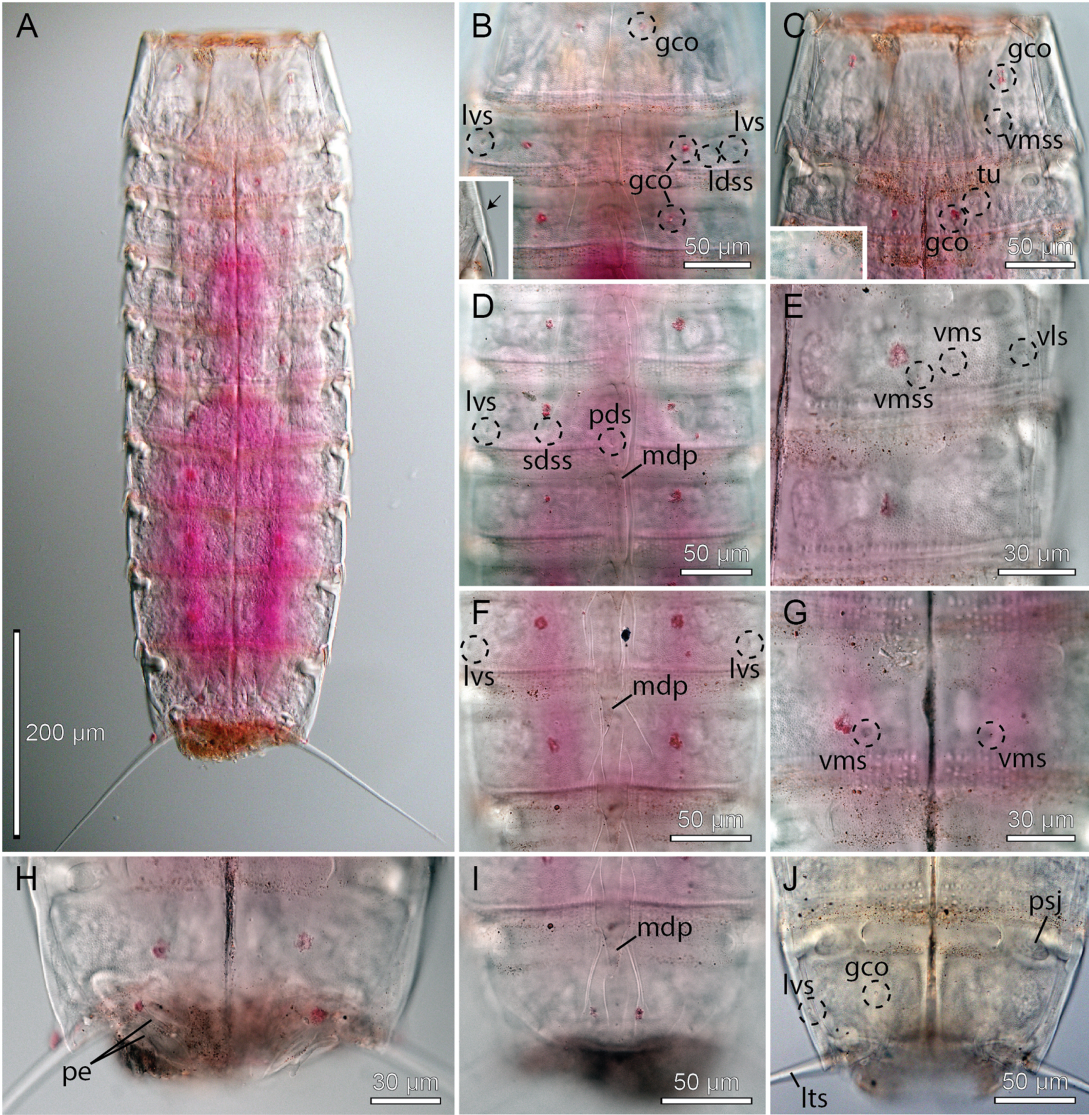
Light micrographs showing overviews and details of male holotype, NHMD-233061 (A–I) and female paratype, NHMD-233063 (J), of *Cristaphyes scatha* sp. nov. Note glandular cell outlets that appear as dots with extra strong Rose Bengal staining. (A) Ventral overview. (B) Segments 1–3, dorsal view; inset shows paralateral seta (arrow). (C) Segments 1–2, ventral view; inset shows ventromedial male tube. (D) Segments 5–7, dorsal view. (E) Segments 5–6, left side sternal plates. (F) Segments 8–9, dorsal view. (G) Segment 9, ventromedial parts of sternal plates. (H) Segments 10–11 showing male morphology, ventral view. (I) Segments 10–11, dorsal view. (J) Segments 10–11, showing female morphology, ventral view. Abbreviations: gco, glandular cell outlet; ldss, laterodorsal sensory spot; lts, lateral terminal spine; lvs, lateroventral seta; mdp, middorsal process; pds, paradorsal seta; pe, penile spines; psj, peg-and-socket joint; sdss, subdorsal sensory spot; tu, tube; vls, ventrolateral seta; vms, ventromedial seta; vmss, ventromedial sensory spot.

**Figure 12:**
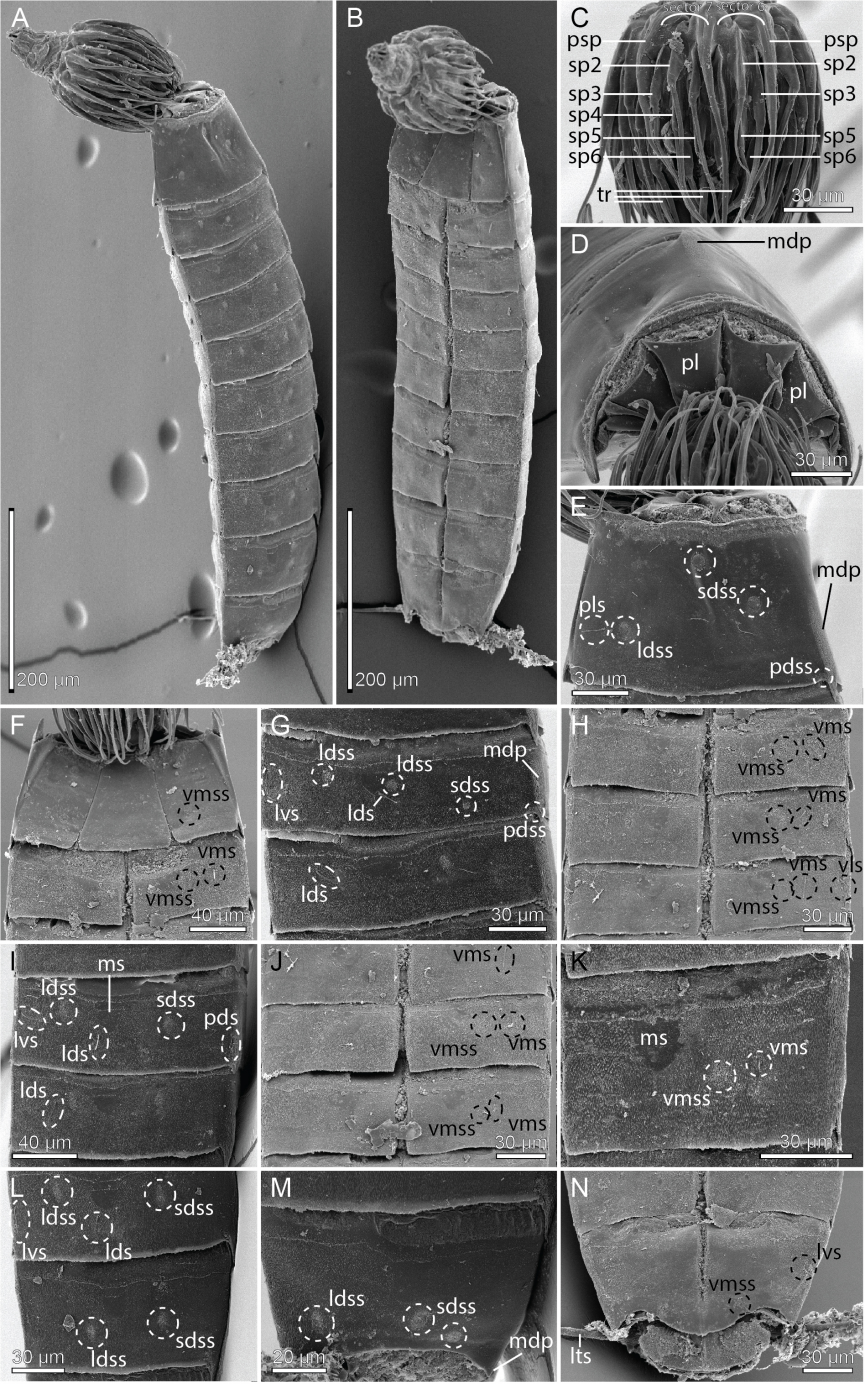
Scanning electron micrographs showing overviews and details of female *Cristaphyes scatha* sp. nov. (A) Left lateral overview. (B) Ventrolateral overview. (C) Detail of head showing introvert sectors 6 and 7. (D) Segment 1, frontal view. (E) Segment 1, left side tergal plate. (F) Segments 1 and 2, ventral view. (G) Segments 2 and 3, left side tergal plates. (H) Segment 3–5, ventral view. (I) Segments 6–7, left side tergal plates. (J) Segment 5–7, ventral view. (K) Segment 8, left side sternal plate. (L) Segments 8–9, left side tergal plates. (M) Segment 10, left side tergal plate. (N) Segments 9–11, ventral view. Abbreviations: lds, laterodorsal seta; ldss, laterodorsal sensory spot; lts, lateral terminal spine; lvs, lateroventral seta; mdp, middorsal process; ms, muscular scar; pds, paradorsal seta; pdss, paradorsal sensory spot; pl, placid; pls, paralateral seta; psp, primary spinoscalid; sdss, subdorsal sensory spot; sp, spinoscalid followed by introvert ring number; tr, trichoscalid; vls, ventrolateral seta; vms, ventromedial seta; vmss, ventromedial sensory spot.

urn:lsid:zoobank.org:act:4634228E-EB90-424F-A99A-6519D189B30C
Figures 9–12, Tables 6 and 7

### Diagnosis

*Cristaphyes* with middorsal processes on segments 1–10, with the process of segment 10 very short, not projecting beyond the terminal segment. Setae present in: paradorsal position on segments 4 and 6 (unpaired, single setae on both segments), laterodorsal positions of segments 2–8 (but alternating between very laterally displaced setae on uneven numbered segments, and setae close to the subdorsal region on even numbered segments), paralateral positions of segment 1, lateroventral positions of segments 2, 4, 6, 8, and 10, ventrolateral positions on segment 5, and ventromedial positions on segments 2–9 in females and 3–9 in males (setae on segment 9 displaced and very close to the paraventral region). Males with ventromedial tubes on segment 2. Lateral terminal spines present.

### Etymology

The species is named *scatha*, after Scatha—one of the few surviving dragons bred by Morgoth during the First Age—in the book Silmarillion by JRR Tolkien.

### Material examined

Holotype, adult male, collected from mud on August 7, 2013, on St. KG1 at 105 m depth in Kongsfjorden (78°55.85′N 012°08.37′E), mounted in Fluoromount G, deposited at the Natural History Museum of Denmark, under catalogue number NHMD-233061. Paratypes include one female from same locality as the holotype, and one most likely preadult (J6) female, collected from mud on August 5, 2013, on St. KB2 at 310 m depth in Kongsfjorden (78°58.69′N 011°42.79′E), both mounted in Fluoromount G, and deposited at the Natural History Museum of Denmark, under catalogue numbers NHMD-233062 to 233063. Additional non-type specimens include one adult female, collected from mud on May 21, 2016, on St. A3 at 96 m depth in Storfjorden (77°56.61′N 020°13.10′E), mounted for SEM and stored in the first author’s personal reference collection. See Fig. 1 for localities and Table 1 for detailed station data.

### Description

Adults with head, neck, and eleven trunk segments (Figs. 9A–9B, 10, 11A and 12A–12B). The trunk is nearly parallel sided from segments 1 to 9. The terminal segment is almost completely covered by segment 10. Segment 1 consists of a tergal, two episternal and a midsternal plate (Figs. 9B, 11C and 12F), whereas the following ten segments consist of a tergal and two sternal plates. Lateral terminal spines are present, and about the same length as segments 8–10. For complete overview of measures and dimensions, see Table 6. Distribution of cuticular structures, that is, sensory spots, tubes and setae, is summarized in Table 7.

**Table 6:** Measurements from light microscopy of *Cristaphyes scatha* sp. nov. (in μm).

Character	♂ Holotype	♀ Paratype	♀ Paratype
	NHMD-233061	NHMD-233062	NHMD-233063
TL	717	744	759
MSW-6	203	199	208
MSW-6/TL	28.3%	26.7%	27.4%
SW-10	169	159	178
SW-10/TL	23.6%	21.4%	23.50%
S1	107	108	110
S2	74	69	70
S3	72	73	79
S4	78	73	78
S5	78	80	81
S6	80	80	82
S7	83	78	81
S8	85	79	87
S9	92	84	94
S10	96	91	99
S11	42	43	51
LTS	212	182	195
LTS/TL	29.6%	24.5%	24.7%

**Note:**

LTS, lateral terminal spine; MSW-6, maximum sternal width, measured on segment 6 in this species; S, segment lengths; SW-10, standard width, always measured on segment 10; TL, trunk length.

**Table 7:** Summary of nature and location of sensory spots, setae, and tubes arranged by series in *Cristaphyes scatha* sp. nov.

Position segment	PD	SD	LD	PL	LV	VL	VM
1	ss	ss, ss	ss	se			ss
2	ss	ss	ss, se, ss		se		se(♀), ss, tu(♂),
3	ss	ss	ss, se				se, ss
4	ss, se*	ss	se, ss		se		se, ss
5	ss	ss	ss, se			se	se, ss
6	ss, se*	ss	se, ss		se		se, ss
7	ss	ss	ss, se				se, ss
8	ss	ss	se, ss		se		se, ss
9	ss	ss	ss			ss	se, ss
10		ss, ss	ss		se		ss
11		ss3			lts	pe, pe(♂)	

**Notes:**

LD, Laterodorsal; LV, lateroventral; PD, paradorsal; PL, paralateral; SD, subdorsal; VL, ventrolateral; VM, ventromedial; lts, lateral terminal spine; pe, penile spines; se, seta; ss, sensory spot, 3 marks type 3 sensory spot; tu, tube; (♂) male and (♀) female conditions of sexually dimorphic characters.

* Marks unpaired setae.

The mouth cone has nine outer oral styles, arranged as one anterior to each introvert sector, expect for the middorsal sector 6 (Fig. 10). Each outer oral style consists of a single flexible unit. Proximally they attach to the mouth cone, but it was difficult to visualize details in the distal part of the mouth cone because this part of the single specimen available for SEM was slightly collapsed.

The introvert is equipped with spinoscalids, arranged in transverse rings and longitudinally in 10 sectors, defined by the primary spinoscalids of ring 01 (Figs. 10 and 12C). The primary spinoscalids consist of a stout proximal unit with a weakly developed median fringe, and a long, slender, end piece (Fig. 12C). Spinoscalids of rings 02 and 03 have more narrow proximal sheaths, with short fringes along their proximal margins. End pieces are long, and more slender than those in ring 01. Spinoscalids of the remaining rings also consist of a proximal sheath and an end piece, but they are shorter. Ring-wise arrangement of spinoscalids is as follows: Ring 01—10 primary spinoscalids; Ring 02—10 spinoscalids, one medially in each sector; Ring 03—20 spinoscalids, one pair in each sector; Ring 04—five spinoscalids, one medially but in uneven-numbered sectors only; Ring 05—15 spinoscalids, one pair in uneven-numbered sectors, and one medially in even-numbered sectors; Ring 06—15 spinoscalids, one medially in uneven-numbered sectors, and a pair in even-numbered sectors (Figs. 10 and 12C). Described sector-wise, all uneven-numbered sectors have seven spinoscalids, arranged as a double diamond. Even-numbered sectors have spinoscalids forming two chevrons (i.e., a single and a pair of spinoscalids in each chevron), with a blank ring separating the two chevrons. A total of 14 trichoscalids are present posterior to the spinoscalid rings. They are located as single trichoscalids in even-numbered sectors, and in sector 1, and as pairs in the remaining uneven-numbered sectors (Fig. 10).

The neck has four dorsal and two ventral placids (Figs. 9 and 12D); all placids are rectangular and measures in width: 42 μm (subdorsal pair) and 33 μm (laterodorsal and ventral pairs), respectively.

Middorsal processes are present on segments 1–10; processes on segments 1–9 project only slightly beyond the posterior segment margins, whereas the process on segment 10 is even shorter (Figs. 9A, 11D, 11I, 12A, 12D–12E, 12G, 12I and 12L–12M). Rounded to oval glandular cell outlets are present in series on the dorsal and ventral sides, in subdorsal positions on segments 1 and 10, in laterodorsal positions in segments 2–9, in ventromedial positions on segments 1 and 3–10, and in paraventral positions on segment 2 (Figs. 9A–9B, 11B–11C and 11J). Smooth, hairless areas (muscle scars) marking subcuticular muscle attachment sites are present anteriorly on the segments, in laterodorsal and ventromedial positions (Fig. 12K) on segments 2–9 (Figs. 9A and 9B). All segments, except anterior 3/4 of segment 1, are covered with very minute acicular hairs. Secondary fringes, formed by one or two wavy bands, are present in segments 2–10. Pachycycli, and peg-and-socket joints are present on segments 2–10. Paraventral apodemes are absent on all segments.

Segment 1 with middorsal process, arising on posterior 1/3 of segment, and projecting slightly beyond the posterior segment margin; ridge of process is covered by densely set hairs (Figs. 9A and 12D–12E). Midsternal plate trapezoid (Figs. 9B, 11C and 12F). Anterior segment margin with narrow reticulated area along the margins of the tergal plate, and slightly larger reticulated areas along margins of episternal plates. The segmental plates terminate posteriorly in free flaps, with finely serrated margins. Setae present in paralateral positions (Figs. 9A, 11B inset and 12E). Sensory spots present in paradorsal positions at posterior segment margin near projecting part of middorsal process; and as two pairs in subdorsal positions, one pair medially on segment and the other more anterior; anterior pair appears to be located in the anterior ends of elongated depressed areas in the cuticle (Figs. 9A and 12E). Sensory spots furthermore present in laterodorsal and ventromedial positions, more posteriorly on segment (Figs. 9A–9B, 11C and 12E–12F). All sensory spots, on this and following segments, appear conspicuously large and distinct in this species.

Segment 2 with middorsal process and paradorsal sensory spots as on preceding segment, but with ridge of middorsal process expanding from a more anterior position on the segment (Figs. 9A and 12G). Tergal plate furthermore with setae in laterodorsal (close to subdorsal) and lateroventral positions, one pair of sensory spots in subdorsal positions, and two pairs in laterodorsal positions, flanking the muscle scar and the laterodorsal setae (Figs. 9A, 11B and 12G). Sternal plates with ventromedial sensory spots in both sexes; females furthermore with ventromedial (very close to ventrolateral) setae (Figs. 9C and 12F), and males with ventromedial tubes (Figs. 9B and 11C inset). Posterior segment margin as on preceding segment.

Segment 3 with middorsal process and paradorsal sensory spots as on preceding segment. Tergal plate furthermore with subdorsal and laterodorsal sensory spots, and laterodorsal setae located much more lateral than those on segment 2 (Figs. 9A, 11B and 12G). Sternal plates with ventromedial setae and sensory spots (Figs. 9B and 12H).

Segment 4 with middorsal process and paradorsal sensory spots as on preceding segment. Tergal plate furthermore with unpaired seta in paradorsal position, paired laterodorsal, and lateroventral setae (laterodorsal ones located more dorsal than those on preceding segment), and sensory spots in subdorsal and laterodorsal positions (Fig. 9A). Sternal plates as on preceding segment (Figs. 9B and 12H).

Segment 5 similar to segment 3, but with the addition of a pair of ventrolateral setae (Figs. 9A–9B, 11D–11E, 12H and 12J).

Segment 6 similar to segment 4 (Figs. 9A–9B, 11D–11E and 12I–12J).

Segment 7 similar to segment 3, except for the ventromedial setae being located closer to the ventromedial sensory spots (Figs. 9A–9B and 12I–12J).

Segment 8 similar to segments 4 and 6, except for the absence of paradorsal setae (Figs. 9A–9B, 11F and 12K–12L). Setae on the sternal plates are ventromedial, as on preceding segments, but laterally displaced and very close to ventrolateral positions.

Segment 9 with middorsal process, flanked by paradorsal sensory spots. Tergal plate otherwise with sensory spots in subdorsal and laterodorsal positions (Figs. 9A, 11F and 12L). Sternal plates with ventrolateral sensory spots, and ventromedial setae and sensory spots, but with the setae located very close to the paraventral region, closer to the midventral line than the sensory spots (Figs. 9B, 9D, 11G and 12N).

Segment 10 with very short middorsal process without conspicuous middorsal ridge and flanking sensory spots, not projecting beyond the terminal segment (Figs. 9A, 11J, 12M and 12N). Tergal plate otherwise with lateroventral setae, and two pairs of sensory spots in subdorsal positions and one pair in laterodorsal positions (all near posterior segment margin) (Figs. 9A, 11I and 12M). Sternal plates with ventromedial sensory spots, near posterior segment margin (Figs. 9B, 9D, 11J and 12N). Posterior margin of tergal plate is straight as on all preceding segment, whereas the margins of the sternal plates are concave, and similar in both sexes. Posterolateral corners of tergosternal junctions form slightly pointed caudal projections.

Segment 11 hardly projecting beyond segment 10. Lateral terminal spines present (Figs. 9A–9B, 11A and 11J). Tergal plate with a pair of slightly projecting type 3 sensory spots in subdorsal positions. Margin of sternal plates with pair of pointed, ventromedial horn-like projections. Two pairs of penile spines present in males (Figs. 9B and 11H).

### Remarks for *Cristaphyes scatha* sp. nov.

*Cristaphyes scatha* sp. nov. is easily distinguished from the eight congeners without lateral terminal spines. The species’ very short middorsal process on segment 10 also distinguishes it from congeners with a longer process that projects beyond the terminal segment. These include *C. abyssorum*, *C. arctous*, *C. chukchiensis*, *C. cristatus*, *C. dordaidelosensis* sp. nov., *C. furugelmi*, *C. glaurung* sp. nov., and *C. nubilis* (Higgins, 1991; Adrianov & Malakhov, 1999; Sánchez et al., 2013; Sánchez, Pardos & Sørensen, 2014; Adrianov & Maiorova, 2015). The remaining five congeners without or only with very short, non-projecting middorsal process on segment 10 include *C. carinatus*, *C. chilensis*, *C. cryopygus*, *C. longicornis*, and *C. odhneri. C. carinatus* is quite easily distinguished from its congeners by its anteriorly extended midsternal plate on segment 1 that projects beyond the anterior margins of its episternal plates (Zelinka, 1928). The description of *C. chilensis* does not provide any consistent information about distribution of setae, but the species is easily distinguished from most of congeners by the broadly pointed posterior tergal margin of segment 10 (Lang, 1953). Segment 10 of *C. scatha* sp. nov. also has a small, middorsal, posterior process, but the sides of the process only expand from the paradorsal positions. The edges of the middorsal posterior point or process in *S. chilensis* starts expanding almost from the laterodorsal positions.

The remaining three species are mainly distinguished by their distribution of setae. Information on seta distribution in *C. odhneri* provided by Lang (1949) is probably not complete (no dorsal setae are reported), but the illustrations of Lang (1949) clearly indicate that setae on the sternal plates are located very medial on each plate, or closest to the midventral line. In *C. scatha* sp. nov. the ventromedial setae are much more laterally displaced, and are located very close to the ventrolateral areas. Both *C. cryopygus* and *C. longicornis* are distinguished from *C. scatha* sp. nov. by the distribution pattern of their setae on the tergal plates. *C. cryopygus* has longitudinally aligned laterodorsal setae on segments 2, 4, and 6–10, lateroventral setae on segments 2 and 10 (Higgins & Kristensen, 1988), and no paralateral setae on segment 1. On the opposite, *C. scatha* sp. nov. has laterodorsal setae on segments 2–8 that alternate between more dorsal (even numbered segments) and more lateral (uneven-numbered segments) positions, lateroventral setae in even-numbered segments only, and presence of paralateral setae on segment 1. *C. scatha* sp. nov. shows the greatest resemblance with *C. longicornis*, described from Belize (Higgins, 1983). Both species have paralateral setae on segment 1, laterodorsal setae on segments 2–8, and lateroventral setae on even numbered segments only. However, *C. longicornis* has laterodorsal setae on segment 9 also (missing in *C. scatha* sp. nov.), and its laterodorsal setae are all aligned, opposed to the laterodorsal setae in *C. scatha* sp. nov. that shifts positions. The episternal plates of *C. longicornis* furthermore have a seta each, whereas such setae are missing in *C. scatha* sp. nov., and finally males of *C. longicornis* do not have ventromedial tubes on segment 2.

**Figure 13:**
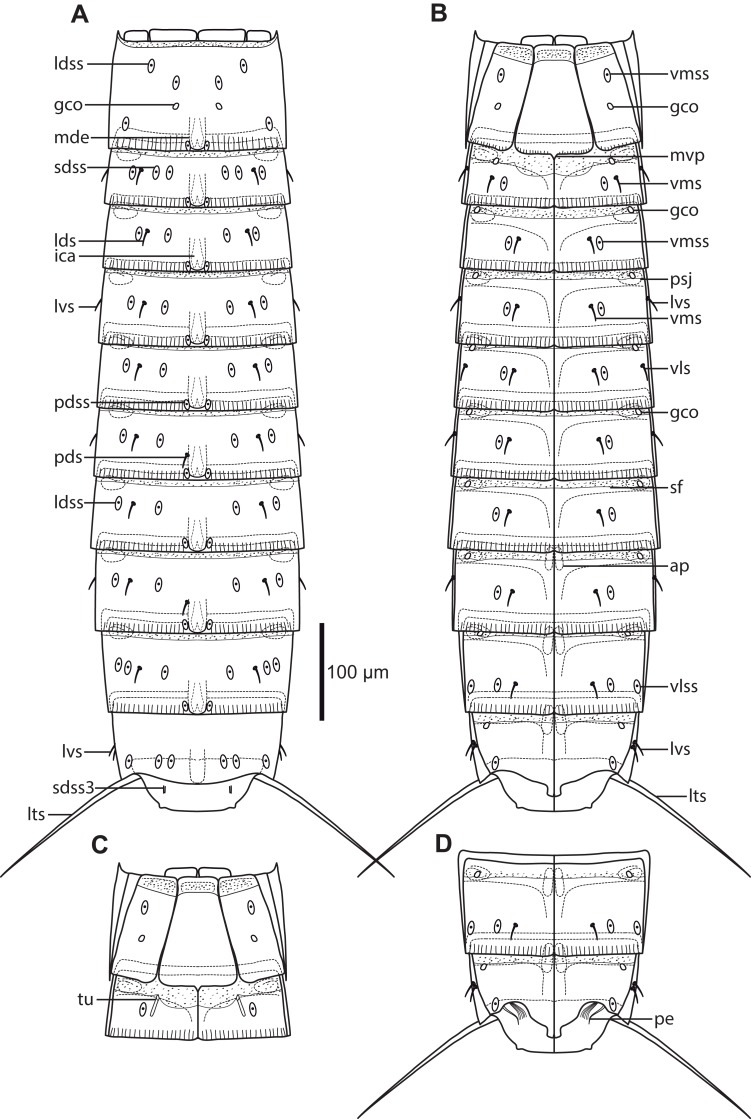
Line art illustrations of *Pycnophyes ancalagon* sp. nov. (A) Female, dorsal view. (B) Female, ventral view. (C) Male, segments 1–2, ventral view. (D) Male, segments 9–11, ventral view. Abbreviations: ap, apodeme; gco, glandular cell outlet; ica, intracuticular atria; lds, laterodorsal seta; ldss, laterodorsal sensory spot; lts, lateral terminal spine; lvs, lateroventral seta; mde, middorsal elevation; mvp, midventral process; pds, paradorsal seta; pdss, paradorsal sensory spot; pe, penile spines; psj, peg-and-socket joint; sdss, subdorsal sensory spot; sdss3, subdorsal sensory spot type 3; sf, secondary fringe; tu, tube; vls, ventrolateral seta; vlss, ventrolateral sensory spot; vms, ventromedial seta; vmss, ventromedial sensory spot.

**Figure 14:**
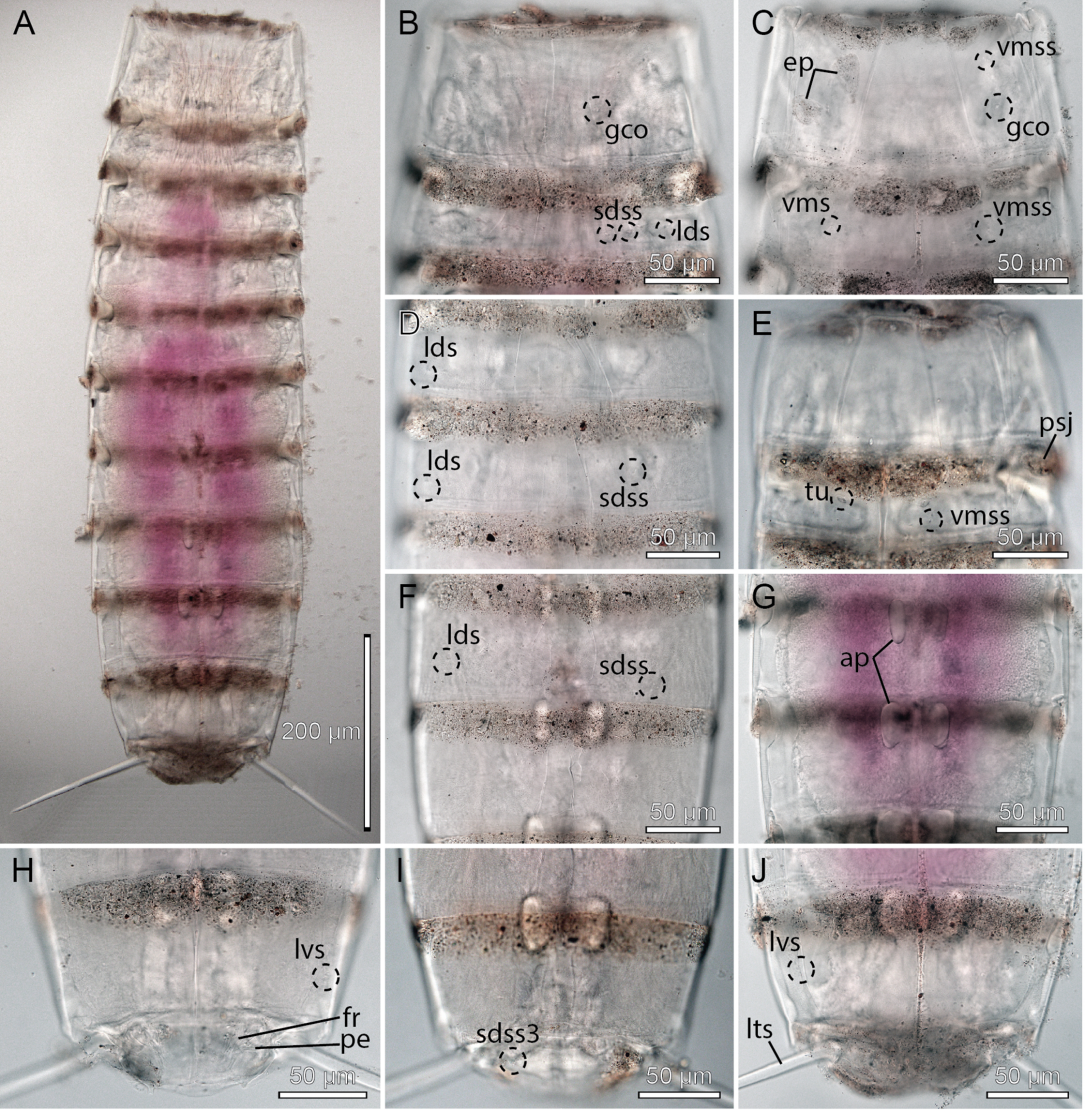
Light micrographs showing overviews and details of female holotype, NHMD-233064 (A–C, G, J) and male paratype, NHMD-233072 (D–F, H–I), of *Pycnophyes ancalagon* sp. nov. (A) Ventral overview. (B) Segments 1–2, dorsal view. (C) Segments 1–2 showing female morphology, ventral view. (D) Segments 5–6, dorsal view. (E) Segments 1–2 showing male morphology, ventral view. (F) Segments 7–8, dorsal view. (G) Segments 8–9, ventral view. (H) Segments 10–11 showing male morphology, ventral view. (I) Segments 10–11, dorsal view. (J) Segments 10–11, showing female morphology, ventral view. Abbreviations: ap, apodeme; ep, epibiont; fr, fringe; gco, glandular cell outlet; lds, laterodorsal seta; lts, lateral terminal spine; lvs, lateroventral seta; pe, penile spine; psj, peg-and-socket joint; sdss, subdorsal sensory spot; sdss3, subdorsal sensory spot type 3; tu, tube; vms, ventromedial seta; vmss, ventromedial sensory spot.

**Figure 15:**
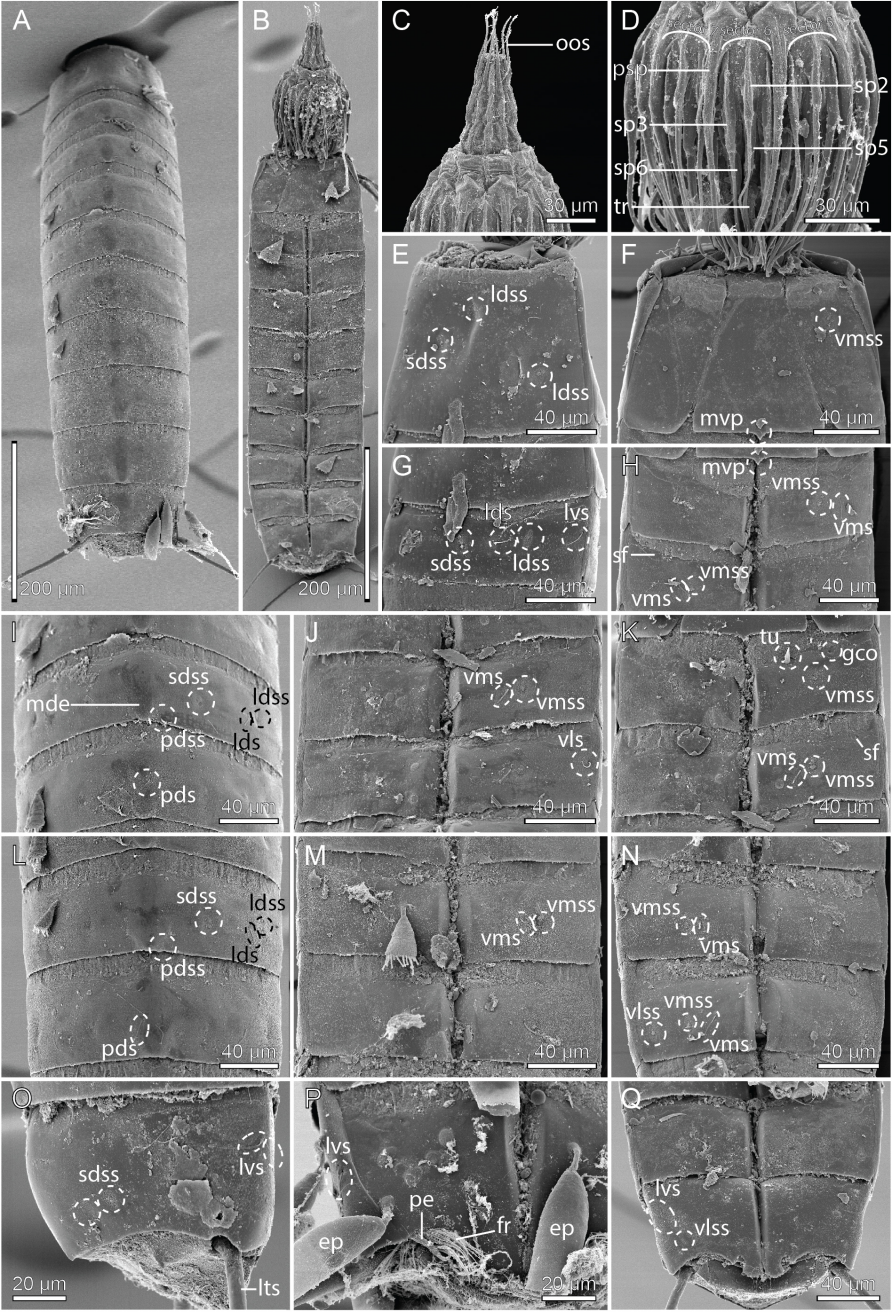
Scanning electron micrographs showing overviews and details of *Pycnophyes ancalagon* sp. nov. (A) Dorsal overview. (B) Ventral overview. (C) Detail of head showing mouth cone with outer oral styles. (D) Detail of head showing introvert sectors 5–7. (E) Segment 1, right side tergal plate. (F) Segment 1, ventral view. (G) Segment 2, right side tergal plate. (H) Segment 2–3 in female, ventral view. (I) Segments 5–6, dorsal view. (J) Segments 4–5, ventral view. (K) Segment 2–3 in male, ventral view. (L) Segments 7–8, dorsal view. (M) Segments 6–7 ventral view. (N) Segments 8–9, ventral view. (O) Segments 10–11 in female, right side tergal plates. (P) Segment 10 in male, right side sternal plate. (Q) Segments 9–11 in female, ventral view. Abbreviations: ep, epibiont; fr, fringe; gco, glandular cell outlet; lds, laterodorsal seta; ldss, laterodorsal sensory spot; lts, lateral terminal spine; lvs, lateroventral seta; mde, middorsal elevation; mvp, midventral process; oos, outer oral style; pds, paradorsal seta; pdss, paradorsal sensory spot; pe, penile spine; psp, primary spinoscalid; sdss, subdorsal sensory spot; sf, secondary fringe; sp, spinoscalid followed by introvert ring number; tr, trichoscalid; tu, tube; vls, ventrolateral seta; vlss, ventrolateral sensory spot; vms, ventromedial seta; vmss, ventromedial sensory spot.

Genus *Pycnophyes* Zelinka, 1907
***Pycnophyes ancalagon* sp. nov.**
urn:lsid:zoobank.org:act:47580836-3AF4-4273-89DA-C0B86E84AF69
Figs. 13–15, Tables 8 and 9

### Diagnosis

*Pycnophyes* with middorsal elevations and intracuticular atria on segments 1–9. Posterior margin of midsternal plate of segment 1 with short, pointed midventral process. Setae present in: paradorsal position on segments 6 and 8 (unpaired, single setae on both segments), laterodorsal positions of segments 2–9, lateroventral positions of segments 2, 4, 6, 8 and 10 (twin pair on segment 10), ventrolateral positions on segment 5, and ventromedial positions on segments 2–9 in females and 3–9 in males. Males with ventromedial tubes on segment 2, and a single pair of penile spines of segment 10. Lateral terminal spines present.

### Etymology

The species is named *ancalagon*, after Ancalagon—the greatest and most powerful of all dragons, bred by Morgoth during the First Age—in the book Silmarillion by JRR Tolkien.

### Material examined

Holotype, adult female, collected from mud on August 7, 2013, on St. KG1 at 105 m depth in Kongsfjorden (78°55.85′N 012°08.37′E), mounted in Fluoromount G, deposited at the Natural History Museum of Denmark, under catalogue number NHMD-233064. Paratypes include four females and two males from same locality as the holotype and one female and one male from St. KB2, also in Kongsfjorden, mounted in Fluoromount G, and deposited at the Natural History Museum of Denmark, under catalogue numbers NHMD-233065 to 233072. Additional non-type specimens include one female and one male from same locality as holotype, and two females and one male, collected from mud on May 21, 2016, on St. A3 at 96 m depth in Storfjorden (77°56.61′N 020°13.10′E), mounted for SEM and stored in the first author’s personal reference collection. See Fig. 1 for localities and Table 1 for detailed station data.

### Description

Adults with head, neck, and eleven trunk segments (Figs. 13A–13B, 14A and 15A–15B). The trunk is nearly parallel sided from segments 1 to 9. Segment 1 consists of a tergal, two episternal and a midsternal plate (Figs. 13B–13C, 14C and 15F), whereas the following 10 segments consist of a tergal and two sternal plates. Lateral terminal spines are present, and about the same length as segments 8–10. For complete overview of measures and dimensions, see Table 8. Distribution of cuticular structures, that is, sensory spots, tubes, and setae, is summarized in Table 9.

**Table 8:** Measurements from light microscopy of *Pycnophyes ancalagon* sp. nov. (in μm), including number of measured specimens (*n*) and standard deviation (SD).

Character	*n*	Range	Mean	SD
TL	9	781–860	817	25.23
MSW-7	9	181–209	199	8.71
MSW-7/TL	9	22.0–26.1%	24.4%	1.25%
SW-10	9	160–176	170	5.22
SW-10/TL	9	19.8–22.3%	20.9%	0.86%
S1	9	104–116	111	3.45
S2	9	74–83	80	2.95
S3	9	75–89	80	4.03
S4	9	80–95	89	5.20
S5	9	85–96	91	3.47
S6	9	87–95	91	2.83
S7	9	87–101	93	3.83
S8	9	93–105	99	4.28
S9	9	96–103	99	2.74
S10	9	82–96	89	4.76
S11	9	51–63	56	4.26
LTS	9	150–205	175	18.22
LTS/TL	9	18.5–25.2%	21.5%	2.30%

**Note:**

LTS, lateral terminal spine; MSW-7, maximum sternal width, measured on segment 7 in this species; S, segment lengths; SW-10, standard width, always measured on segment 10; TL, trunk length.

**Table 9:** Summary of nature and location of sensory spots, setae, and tubes arranged by series in *Pycnophyes ancalagon* sp. nov.

Position segment	PD	SD	LD	LV	VL	VM
1	ss	ss	ss, ss			ss
2	ss	ss, ss	se, ss	se		se(♀), ss, tu(♂),
3	ss	ss	se, ss			ss, se*
4	ss	ss	se, ss	se		ss, se
5	ss	ss	se*, ss		se	ss, se
6	ss, se^#^	ss	se, ss	se		ss, se
7	ss	ss	se, ss			ss, se^¤^
8	ss, se^#^	ss	se, ss	se		ss, se
9	ss	ss	se*, ss, ss		ss	ss, se
10		ss, ss	ss	se, se	ss	
11		ss3		lts	pe(♂)	

**Notes:**

LD, Laterodorsal; LV, lateroventral; PD, paradorsal; SD, subdorsal; VL, ventrolateral; VM, ventromedial; lts, lateral terminal spine; pe, penile spine; se, seta; ss, sensory spot, 3 marks type 3 sensory spot; tu, tube; (♂) male and (♀) female conditions of sexually dimorphic characters.

* Marks setae and sensory spot switch position in some specimens.

¤ Marks that position of seta differs from close to the sensory spot to nearly paraventral.

# Marks that the seta is unpaired.

The mouth cone has nine outer oral styles, arranged as one anterior to each introvert sector, except for the middorsal sector 6. Each outer oral style consists of a single, rather flexible unit (Fig. 15C). Proximally they attach to a smooth mouth cone without any other conspicuous structures.

The introvert is equipped with spinoscalids, arranged in transverse rings and longitudinally in 10 sectors, defined by the primary spinoscalids of ring 01. The primary spinoscalids consist of a stout proximal unit with a median fringe, and a long, slender, end piece (Fig. 15D). Spinoscalids of rings 02 and 03 have proximal sheaths similar to those on the primary spinoscalids, but thinner and shorter end pieces. Spinoscalids of the remaining rings also consist of a proximal sheath and an end piece, but they are even shorter, and without a median fringe. Ring-wise arrangement of spinoscalids is as follows (identical with the arrangement in *C. scatha* sp, nov., hence see Fig. 10): Ring 01—10 primary spinoscalids; Ring 02—10 spinoscalids, one medially in each sector; Ring 03–20 spinoscalids, one pair in each sector; Ring 04—five spinoscalids, one medially but in uneven-numbered sectors only; Ring 05—14 spinoscalids, one pair in uneven-numbered sectors, and one medially in even-numbered sectors, except the middorsal sector 6; Ring 06—14 spinoscalids, one medially in uneven-numbered sectors and sector 6, and one pair in remaining sectors. Described sector-wise, all uneven-numbered sectors have seven spinoscalids, arranged as a double diamond. Even-numbered sectors, except sector 6, have spinoscalids forming two chevrons (i.e., a single and a pair of spinoscalids in each chevron), with a blank ring separating the two chevrons. Sector 6 has a chevron in Rings 02–03, and then a single, medial spinoscalid in Ring 06 (Figs. 10 and 15D). Fourteen trichoscalids are present posterior to the spinoscalid rings. They are located as single trichoscalids in even-numbered sectors, and in sector 1, and as pairs in the remaining uneven-numbered sectors.

The neck has four dorsal and two ventral placids; all placids are rectangular and measures in width: 50 μm (subdorsal pair), 24 μm (laterodorsal pair), and 30 μm (ventral pair).

Middorsal elevations with intracuticular atria are present on segments 1–9 (Figs. 13A, 15A, 15I and 15L); segment 10 has a weak elevation only, but no intracuticular structures. The cuticle appears rather thin, which makes it harder to visualize intracuticular structures in LM. Glandular cell outlets are present in series on the ventral side, in ventromedial positions on segments 1 and 2, and in ventrolateral positions on segments 3–10 (Figs. 13A–13B, 14C and 15K); glandular cell outlets on segment 1 are located medially on episternal plates, whereas those on segments 2–10 are located anteriorly on segment, at the rim of the secondary fringe (Fig. 15K). On the dorsal side, glandular cell outlets were only identified in subdorsal positions on segment 1 (Fig. 14B). Muscle attachment sites (muscular scars) are likewise weakly defined, and were not identified. Paired paraventral apodemes are present on segments 8–10 (Figs. 13B and 14F–14J), and in two specimens on segment 7 as well; apodemes are largest on the most posterior segments. Segment 1 is nearly completely smooth (Fig. 15E), whereas segments 2–10 have minute, scale-like cuticular hairs. Secondary fringes, formed by one to two bands, are present in segments 2–10; the secondary fringe is broad and lobed on the ventral side of segment 2, but narrower dorsally, and on the remaining segments. Pachycycli, and peg-and-socket joints are present on segments 2–10.

Segment 1 with trapezoid midsternal plate with short pointed midventral process (Fig. 15F). Anterior segment margin with narrow reticulated area along the margins of the tergal plate, and slightly larger depressed and reticulated areas along margins of the midsternal and episternal plates (Figs. 15E and 15F). The segmental plates terminate posteriorly in free flaps, with finely serrated margins. Setae are absent. Sensory spots present on posterior part of tergal plate in paradorsal and laterodorsal positions, and on anterior part of plate in subdorsal and laterodorsal positions (Figs. 13A and 15E). Episternal plates with one pair of ventromedial sensory spots on anterior parts of plates (Figs. 13B, 14C and 15F).

Segment 2 with tergal setae in laterodorsal and lateroventral positions, one pair of sensory spots in paradorsal positions, two pairs in subdorsal positions, and one pair in laterodorsal positions, more lateral than the setae (Figs. 13A, 14B and 15G). Sternal plates with ventromedial sensory spots in both sexes; females furthermore with ventromedial setae (Figs. 13B, 14C and 15H), and males with ventromedial tubes (Figs. 13C, 14E and 15K). Posterior segment margin as on preceding segment.

Segment 3 with tergal setae in laterodorsal positions, and sensory spots in paradorsal, subdorsal and laterodorsal positions (Fig. 13A). Sternal plates with ventromedial setae and sensory spots (Figs. 9B and 15H); setae are most commonly closest to the midventral line, but in one specimen they had switched positions.

Segment 4 with tergal plate similar to segment 3, except for the addition of a pair of lateroventral setae (Fig. 13A). Sternal plates as on preceding segment, but with setae always appearing closest to the midventral line (Figs. 13B and 15J).

Segment 5 with tergal similar to that of segment 3, but with laterodorsal setae and sensory spots switching position in some specimens (Figs. 13A, 14D and 15I). Sternal plates identical with segment 4, but with the addition of a pair of ventrolateral setae (Figs. 13B and 15J).

Segment 6 with unpaired seta in paradorsal position (Figs. 13A and 15I). Segment otherwise similar to segment 4.

Segment 7 with tergal plate similar to segments 3 and 5 (Figs. 13A, 14F and 15L), and sternal plates similar to with preceding segment (Figs. 13B and 15M); the exact position of the ventromedial setae differs between the specimens (independent of sex), and appear in some specimens very close to the paraventral areas.

Segment 8 similar to segment 6, inclusive the paradorsal seta (Figs. 13A–13B, 14G and 15N).

Segment 9 with tergal setae in laterodorsal positions, and one pair of sensory spots in paradorsal and subdorsal positions, and two pairs in laterodorsal positions (Fig. 13A); in most specimens both laterodorsal sensory spots are located lateral to the setae, but in a single specimen, one pair of sensory spots is more dorsal, located very close to the subdorsal positions. Sternal plates with setae in ventromedial positions, and sensory spots in ventrolateral and ventromedial positions (Figs. 13B, 13D and 15N).

Segment 10 with two pairs of very closely set lateroventral setae (Figs. 13A–13B, 13D, 14H, 13J, 15O–15Q); no other setae are present on the segment. Sensory spots are present as two pairs in subdorsal positions, one pair in laterodorsal positions, and one pair in ventrolateral positions, all close to the posterior segment margin (Figs. 13A–13B, 13D, 15O and 15Q). Posterior margin of tergal plate is slightly convex, whereas the margins of the sternal plates are concave with narrow extensions near the midventral articulation. Posterolateral corners of tergosternal junctions form slightly pointed caudal projections.

Segment 11 projecting beyond segment 10. Lateral terminal spines present (Figs. 13A–13B and 14A). Tergal plate with pair of type 3 sensory spots in subdorsal positions (Fig. 14I). Margin of sternal plates with pair of short, truncate projections. Males apparently only with a single pair of penile spines, lateral to a tuft of long fringe-like extensions (Figs. 13D, 14H and 15P).

### Epibiontic growth

All examined specimens carried one to numerous unidentified loricate, protist (most likely ciliate) epibionts (Figs. 14C, 15A–15B, 15M and 15P). The colonization is noteworthy because none of the examined species of *Cristaphyes* carried any epibionts. This suggests that the epibionts show a genus- or species preference when they infect.

The presence of epibiontic growth on kinorhynchs is not uncommon, and scattered information can be found in the literature (Adrianov & Higgins, 1996; Ostmann, Nordhaus & Sørensen, 2012; Sørensen & Landers, 2017, 2018; Herranz, Yangel & Leander, 2018), even though there have been few attempts to address the phenomenon in general. Amongst species of Pycnophyidae, Neuhaus (2013) reports that sessile epibionts are common on *P. communis* Zelinka, 1928, *P. parasanjuanensis* Adrianov & Higgins, 1996, *Setaphyes dentatus* (Reinhard, 1881), and *Setaphyes kielensis* (Zelinka, 1928). The first author furthermore has unpublished photos of *K. greenlandica*, *Leiocanthus pardosi* (Sánchez et al., 2013), and *P. tubuliferus* Adrianov, 1989 with growth of similar epibionts attached. Since the available reports of epibionts often come without proper identification, it is still way too premature to draw any conclusions about host-symbiont preferences, but the frequent reports of such epibionts suggests that the topic should be addressed in future studies.

### Remarks for *Pycnophyes ancalagon* sp. nov.

The new species fits the emended genus diagnosis of *Pycnophyes* (see Sánchez et al., 2016): middorsal elevations are present on segments 2–9 (excludes *Leiocanthus*), middorsal processes are not present (excludes *Cristaphyes*), paradorsal setae are present on segments 6 and 8 only (excludes *Krakenella* and *Setaphyes*), lateroventral setae are present on even numbered segments (excludes *Setaphyes* and also *Higginsium*), and ventrolateral setae on segment 5 only (excludes *Fujuriphyes*, *Higginsium*, and *Setaphyes*). Sánchez et al. (2016) suggest that the length of the lateral terminal spines never exceeds 20% of the total trunk length in species of *Pycnophyes*. With spine/trunk length ratios of 18.5–25.2%, and an average of 21.5%, *P. ancalagon* sp. nov. is in the upper part of the range, but we still feel it can be justified to assign the new species to this genus.

Besides the new species, *Pycnophyes* currently accommodates 25 species. Sánchez et al. (2016) provide a nearly complete species list for the genus (the questionable species *P. echinoderoides* Zelinka, 1928 is omitted though, but according to Neuhaus (2013) the species is still valid), and only *P. alexandroi* Pardos, Sánchez & Herranz, 2016 has been added to the genus since then. Six of the species are easily distinguished from *P. ancalagon* sp. nov. because they do not have lateral terminal spines. Of the remaining ones, the Mediterranean species *P. echinoderoides* Zelinka, 1928 can also be excluded from the comparison since its description is based on juvenile specimens exclusively, and adult morphology is unknown (Zelinka, 1928). Also the North Atlantic *P. calmani* Southern, 1914 is rather poorly described, but one of the few known characteristics for the species is its short lateral terminal spines, measuring 80–100 μm in length only (Southern, 1914), which distinguishes it from *P. ancalagon* sp. nov. that has 50–100% longer spines.

The single character that distinguishes *P. ancalagon* sp. nov. from its remaining 17 congeners is the presence of unpaired paradorsal setae on segments 6 and 8. This character appears so far to be unique for the new species. But even with this character excluded, the combined distribution pattern of the remaining setae is unique for the species. Especially the distribution of lateroventral setae differs a lot amongst the species. *P. ancalagon* sp. nov. has lateroventral setae exclusively on even numbered segments. This feature is shared with only five other species: *P. aulacodes* Sánchez et al., 2012, *P. communis*, *P. norenburgi* Herranz et al., 2014, *P. tubuliferus* and *Pycnophyes zelinkaei* Southern, 2014. However, *P. aulacodes*, *P. norenburgi*, and *P. tubuliferus* all have paralateral setae on segment 1 (Adrianov & Malakhov, 1999; Sánchez et al., 2011; Herranz et al., 2014), which is usually easy to visualized with SEM and hence distinguishes them from *P. ancalagon* sp. nov. *P. zelinkaei* is also easily distinguished by its special fringe at the posterior margin of its segments, and by its numerous subdorsal setae, especially on the more posterior segments (Southern, 1914; Figures 3D–E in Sánchez et al., 2012). *P. ancalagon* sp. nov. does not have such fringes or subdorsal setae at all. The species that shows closest resemblance with *P. ancalagon* sp. nov. is probably *P. communis*. The distribution of setae in *P. communis* is identical with the one in *P. ancalagon* sp. nov., except for its lack of laterodorsal setae in segment 2 (Zelinka, 1928). However, *P. communis* can also be distinguished from *P. ancalagon* sp. nov. by its projecting posterior margin of segment 10, that nearly extends beyond the terminal segment. The posterior margin of segment 10 in *P. ancalagon* sp. nov. is also convex, but the curve is much broader and span over the whole dorsal side. In *P. communis* the convex extension is longer, but also narrower and span only across the subdorsal to subdorsal regions. *P. communis* furthermore has much shorter lateral terminal spines, measuring only 77–100 μm (Zelinka, 1928).

Another relatively unusual feature in *P. ancalagon* sp. nov. is the double pair of lateroventral setae on segment 10. Also this is shared with five other congeners: *P. beaufortensis* Higgins, 1964, *P. communis*, *P. oshoroensis* Yamasaki, Kajihara & Mawatari, 2012, *P. tubuliferus*, and *P. zelinkaei* (plus some congeners without lateral terminal spines). However, both *P. beaufortensis* and *P. oshoroensis* lack the small midventral process on the midsternal plate, which is present in *P. ancalagon* sp. nov., and they furthermore have paralateral or lateroventral setae on segment 1 (Higgins, 1964; Yamasaki, Kajihara & Mawatari, 2012).

Compared with other pycnophyids from Svalbard, *P. ancalagon* sp. nov. is very easily distinguished from the *Cristaphyes* species by its lack of middorsal processes. Instead, it can more easily be confused with *K. mokievskii* and *K. spitsbergensis* that both are described from Isfjorden on the east coast of Spitsbergen (Adrianov, 1995). However, the same diagnostic features as discussed above are also useful to distinguish *P. ancalagon* sp. nov. from the local *Krakenella* species. None of the species have paradorsal setae, double lateroventral setae on segment 10, or midventral process from the midsternal plate of segment 1. *K. spitsbergensis* furthermore lacks laterodorsal setae on segments 5 and 6 (present in *P. ancalagon* sp. nov.), and *K. mokievskii* has lateroventral setae on segments 2–9 (opposed to lateroventral setae only on even numbered segments in *P. ancalagon* sp. nov.). Hence, it is fairly easy to distinguish *P. ancalagon* sp. nov. from the two species of *Krakenella*.

### Additional unidentified *Pycnophyes* spp.

Pycnophyes sp. 1

A single specimen of *Pycnophyes* sp. 1 was collected from St. H1 (Fig. 1; Table 1). The specimen was mounted for SEM, and shows its dorsal side only. Distribution of setae and sensory spots on its dorsal side basically follow the pattern of *P. ancalagon* sp. nov., except for the presence of two subdorsal rather than laterodorsal sensory spots on segment 9. It also has unpaired paradorsal setae on segments 6 and 8, which suggests that the specimen is identical with *P. ancalagon* sp. nov. However, opposed to the *P. ancalagon* sp. nov. that has no outer middorsal structures on segment 10, the specimen from St. H1 has short, pointy process that extends beyond the posterior margin of the segment. This could be interpreted as a late juvenile trait, but it also puts the identity of the specimen in question. Based on the presence of this process, together with our disability of examining cuticular structures on the ventral side, and the fact that it occurs on a different locality than the other *P. ancalagon* sp. nov., we choose not to assign a species name to this particular specimen.

## Discussion

**Figure 16:**
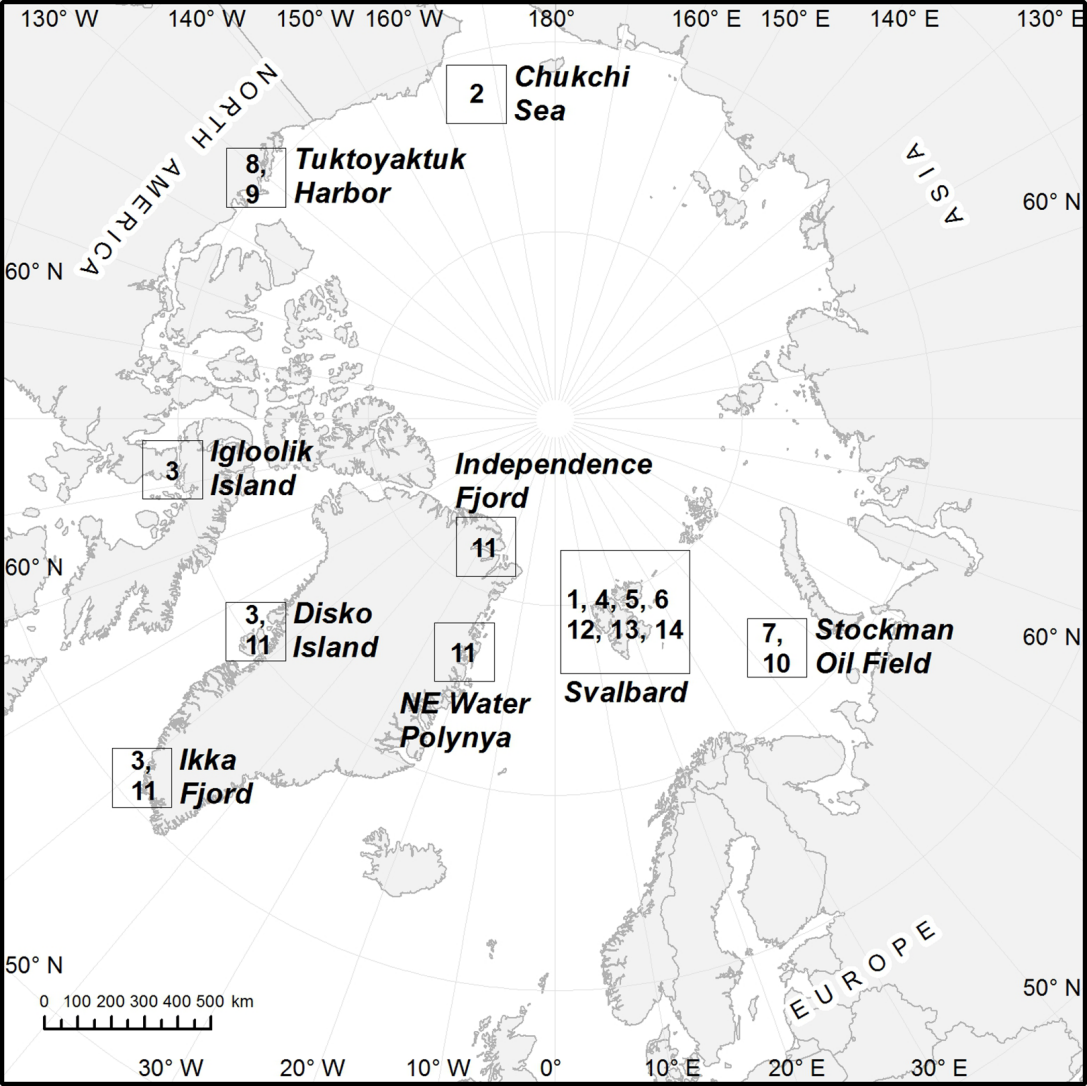
Map showing the distribution of Pycnophyidae in Arctic region. Numbers refer to species names summarized in Table 10.

The descriptions of four new kinorhynch species bring the number of pycnophyid species from Svalbard up to seven, and the total number of Arctic pycnophyids up to 14. A complete summary of all records of pycnophyids in the Arctic, inclusive some previously unpublished records, is presented in Table 10 (see also Fig. 16). This shows that Pycnophyidae is just as diverse in the Arctic as Echinoderidae, which currently is represented with 13 species in the Arctic (Grzelak & Sørensen, in press). Interestingly, Pycnophyidae and Echinoderidae are so far the only two kinorhynch families that have been recorded from the Arctic Region.

**Table 10:** Species of Pycnophyidae from the Arctic region.

	Species name and ID number	Locality	Depth	Source
1	*Cristaphyes arctous*	NE of Svalbard, two localities	345–441 m	Adrianov & Malakhov (1999)
2	*Cristaphyes chukchiensis*	NW of Alaska, Chukchi Sea, several localities	197–210 m	Higgins (1991)
3	*Cristaphyes cryopygus*	Disko Island, West Greenland, several localities	9–300 m	Higgins & Kristensen (1988)
		Igloolik, Nunavut, Canada	67 m	Jørgensen & Kristensen (1991)
		Ikka Fjord, SW Greenland, three localities	19–32 m	NHMD-100126-100131
4	*Cristaphyes dordaidelosensis* sp. nov.	Svalbard, three localities	217–310 m	This study
5	*Cristaphyes glaurung* sp. nov.	Svalbard, six localities	59–310 m	This study
6	*Cristaphyes scatha* sp. nov.	Svalbard, three localities	96–310 m	This study
7	*Krakenella barentsi*	Stockmann Oil Field, Barents Sea	320–340 m	Adrianov & Malakhov (1999)
8	*Krakenella borealis*	Tuktoyaktuk Harbour, NW Territory, Canada, four localities	10–22 m	Higgins & Korczynski (1989)
9	*Krakenella canadensis*	Tuktoyaktuk Harbour, NW Territory, Canada	15 m	Higgins & Korczynski (1989)
10	*Krakenella galtsovae*	Stockmann Oil Field, Barents Sea	320–340 m	Adrianov & Malakhov (1999)
11	*Krakenella greenlandica*	Disko Island, West Greenland, several localities	100–300 m	Higgins & Kristensen (1988)
		Independence Fjord, North Greenland, three localities	24–32 m	NHMD-100088–100089, 100100, 100102–100110, 100151–100161
		North East Water Polynya, Greenland Sea, three localities	105–310 m	NHMD-100090–100099, 100101, 100146–100149
		Ikka Fjord, SW Greenland, three localities	19–32 m	NHMD-100111, 100132–100145
12	*Krakenella mokievskii*	Svalbard	6 m	Adrianov (1995)
13	*Krakenella spitsbergensis*	Svalbard	6 m	Adrianov (1995)
14	*Pycnophyes ancalagon* sp. nov.	Spitsbergen, Svalbard, two localities	96–105 m	This study

**Note:**

ID number refers to species name and corresponds to numbers shown at Fig. 16. NHMD-numbers refer to catalogue numbers of unpublished specimens, stored in the collection of the Natural History Museum of Denmark.

Amongst the pycnophyid genera, *Cristaphyes* and *Krakenella* are the most diverse in the Arctic, with, respectively, six and seven species present. In fact, besides these genera, *P. ancalagon* sp. nov. is the only Arctic species of Pycnophyidae that does not belong to either *Cristaphyes* or *Krakenella*. Considering that the Arctic oceans still remain unexplored to a great degree, it is still way too premature to conclude that *Cristaphyes* and *Krakenella* are more adapted to colonize or specify in Arctic waters, but our current information clearly indicates that this option should be explored further.

Grzelak & Sørensen (in press) suggested that at least some species of Arctic *Echinoderes* appeared to show a circumpolar distribution. A similar distribution pattern is not really clear for any of the recorded pycnophyids. *K. greenlandica* is so far the Arctic pycnophyid that shows the greatest distribution (Table 10), but the species still appears to be restricted to waters around Greenland. Even though species of Pycnophyidae are just as diverse in the Arctic as Echinoderidae, the pycnophyids are usually much less abundant, and it requires a denser and more exhaustive sampling to reveal their true distribution.

## Conclusions

Discovery of four new Arctic mud dragons species from Svalbard demonstrates that the European Arctic represents a region where richness of Kinorhyncha is probably significantly greater than is already known. With the high degree of probability, we can assume that this is also true for other Arctic sectors. An improved sampling and more exhaustive search for Arctic pycnophyids in the future, will hopefully demonstrate their distribution patterns, and show if the species are regionalized or can be found widespread through the Arctic.

## Additional Information and Declarations

### Data Availability

The following information was supplied regarding data availability: All measurements of new species individuals and catalogue numbers of specimens stored in the Natural History Museum of Copenhagen are included in the Results section.

### New Species Registration

The following information was supplied regarding the registration of a newly described species: Publication LSID: urn:lsid:zoobank.org:pub:72D489B2-E8B6-499B-A0C1-10BCCF5E8A29. *Cristaphyes dordaidelosensis* sp. nov.: urn:lsid:zoobank.org:act:12FCA0B9-084A-4C66-841B-186FCC3AFCD0. Cristaphyes glaurung sp. nov.: urn:lsid:zoobank.org:act:7955F387-C093-4823-A416-58A9D2734833. Cristaphyes scatha sp. nov.: urn:lsid:zoobank.org:act:4634228E-EB90-424F-A99A-6519D189B30C. Pycnophyes ancalagon sp. nov.: urn:lsid:zoobank.org:act:47580836-3AF4-4273-89DA-C0B86E84AF69.
